# Non-ambient X-ray and neutron diffraction of novel relaxor ferroelectric *x*Bi_2_(Zn_2/3_,Nb_1/3_)O_3_–(1 – *x*)BaTiO_3_

**DOI:** 10.1107/S160057672100858X

**Published:** 2021-09-29

**Authors:** Jessica Marshall, David Walker, Pamela Thomas

**Affiliations:** a Department of Physics, University of Warwick, Gibbet Hill Road, Coventry, West Midlands CV4 7AL, United Kingdom

**Keywords:** powder neutron diffraction, lead-free relaxors, non-ambient X-ray diffraction, piezoelectricity, lattice parameters

## Abstract

The first determination of the phase diagram of the novel ferroelectric relaxor *x*Bi(Zn_2/3_Nb_1/3_)O_3_–(1 − *x*)BaTiO_3_ (BZN-BT) has been achieved with a combination of high-resolution X-ray and neutron diffraction up to the miscibility limit near *x*(BZN) = 20.0% over a temperature range 20 < *T* < 400 K. The combined X-ray and neutron data show that the instability within the *x*BZN-(1−*x*)BT system reaches a maximum at *x* = 3.9% and is driven by *B*-site displacement and distortion of the oxygen octahedra in the polar phases. Composition-dependent effects include a narrow *Amm*2-dominated region focused at *x* = 3.9%, significant convergence of the lattice parameters in both *P*4*mm* and *Amm*2 phases, and sharp maxima in piezoelectric coefficient *d*_33_ and maximum polarization *P*_max_. Lattice parameter dilation at *x* ≥ 4.0% was observed for both *P*4*mm* and *Amm*2 unit cells, alongside the first appearance of *Pm* 3 *m* at 295 K and the onset of significant dielectric relaxation. Low-temperature neutron diffraction indicated a weak or non-existent temperature dependence on the transition from ferroelectric at *x* = 3.9% to ferroelectric relaxor at *x* = 4.0%. Temperature-dependent phase transitions were eliminated near *x* = 3.0%, with the ferroelectric limit observed at *x* = 5.0% and a transition to a low-loss relaxor dielectric near *x* = 8.0%.

## Abstract 2

Combined non-ambient X-ray and neutron diffraction have been used to observe lattice parameter convergence in polar phases of the *x*Bi(Zn_2/3_ Nb_1/3_)O_3_–(1 − *x*)BaTiO_3_ system below the ferroelectric composition limit at *x* < 5.0%. The lattice parameter convergence is correlated with peaks in the dielectric and piezoelectric coefficients.

## 1. Introduction   

The properties of relaxor ferroelectrics make them useful for a variety of applications including low-loss high-power dielectrics, electrocalorics and nonlinear optical materials (Cross, 1987 ▸; Bokov & Ye, 2006 ▸). Relaxors are characterized by the presence of microscopic clusters a few unit cells in size, known as polar nanoregions (PNRs) (Cross, 1987 ▸; Bokov & Ye, 2006 ▸; Shvartsman *et al.*, 2008 ▸; Shvartsman & Lupascu, 2012 ▸), which (rather than ferroelectric domains) form on cooling through the Burns temperature (*T*_d_). PNRs typically have a lower crystal symmetry than the paraelectric host. They are initially rapidly rotating, weakly coupled polar regions, but on cooling from *T*_d_ to *T*_m_ (defined as the temperature where the frequency-dependent dielectric constant ɛ_m_ is at a maximum), the PNRs slow down, increase in size and show greater coupling to the random fields generated by their polar structure (Cross, 1987 ▸; Bokov & Ye, 2006 ▸; Ge *et al.*, 2013 ▸). The presence of PNRs results in structural distortions within the polar host, often causing multiple polar phases to coexist within relaxor systems at *T* < *T*_m_ (Shvartsman & Lupascu, 2012 ▸; Huband & Thomas, 2017 ▸; Valant, 2012 ▸) and the persistence of microscopic polar phase regions in the paraelectric phase at *T* > *T*_m_. The resulting structural disorder present at the unit-cell level can enhance key ferroelectric properties. Therefore, dopants that form PNRs can be added to classical ferroelectric solid solutions, *e.g.* lead magnesium niobate–lead titanate, lead zinc niobate–lead titanate (PZN-PT) (Nomura *et al.*, 1982 ▸) and sodium bismuth titanate–barium titanate (NBT-BT) (Cross, 1987 ▸; Shvartsman & Lupascu, 2012 ▸; Chen *et al.*, 2014 ▸). Other characteristics of relaxors are diffuse dielectric maxima *ɛ*_m_ and *T*_m_ [*T*_m_ = *T*_c_ (the Curie temperature) for classical ferroelectrics], frequency-dependent *ɛ*_m_ and *T*_m_, and slim hysteresis loops. For relaxors, the dielectric maximum *T*_m_ does not necessarily correspond to a phase change from long-range ferroelectric order to a centrosymmetric paraelectric phase, as is the case for ferroelectric materials at *T* > *T*_c_. A ferroelectric state may form spontaneously at *T* < *T*_m_ or be induced by an applied electric field (Cross, 1987 ▸; Bokov & Ye, 2006 ▸). Recent studies of a lead-free BaTiO_3_-based analogue of PZN-PT, *x*Bi(Zn_2/3_Nb_1/3_)O_3_–(1 − *x*)BaTiO_3_ (BZN-BT) (Ren *et al.*, 2001 ▸; Zhou *et al.*, 2018 ▸; Paterson *et al.*, 2015 ▸), show interesting relaxor properties within the BaTiO_3_ host (Wu *et al.*, 2016 ▸, 2017 ▸; Chen *et al.*, 2015 ▸; Wang *et al.*, 2014 ▸; Marshall *et al.*, 2020 ▸). Previous studies of this system observed the presence of multiple polar phases at 295 K (Wu *et al.*, 2017 ▸; Marshall *et al.*, 2020 ▸), alongside maxima in dielectric constants and the piezoelectric coefficient *d*_33_ near *x* = 4.0%

In the present article, the structural distortions within dilute *x*BZN-(1−*x*)BT (3.0 < *x* < 8.0%) are investigated in detail using temperature-controlled powder X-ray and neutron diffraction. Distortions in the crystal structure and atom displacements are found to be correlated with observed changes in dielectric properties and phase polymorphism in the BZN-BT system. Non-ambient diffraction enables the determination of the phase diagram of the BZN-BT system for 0.0 < *x* < 20.0% from 20 to 400 K for the first time.

## 2. Bi(Zn_2/3_,Nb_1/3_)O_3_–BaTiO_3_: a new lead-free ferroelectric relaxor   

The novel *x*BZN-(1−*x*)BT relaxor system has attracted attention as a high-energy fatigue-resistant capacitor material owing to its low-loss relaxor dielectric nature near *x* = 15.0% (Wu *et al.*, 2016 ▸, 2017 ▸), with a relaxor ferroelectric phase present for *x* < 5.0%. More recent work observed peaks in dielectric and piezoelectric coefficients in the region 3.8 < *x* < 4.0%, which coincided with an *Amm*2-dominated crystal structure at 295 K (Marshall *et al.*, 2020 ▸). This narrow region was also marked by a peak in piezoelectric coefficient with *d*_33_ = 110 pC N^−1^ and a maximum polarization of 26 µC cm^−2^ at *x* = 3.9%, alongside local minima in the peak widths of the (111)_pc_ and (200)_pc_ reflections. The piezoelectric coefficients declined sharply for *x* ≥ 4.0%, and this coincided with changes in the conductivity of *x*BZN-(1−*x*)BT ceramics with composition. For 0 < *x* < 3.9%, the conductance exhibited a local maximum at *T*_m_. Subsequent BZN addition for *x* ≥ 4.0% caused the peak conductance to shift from *T*_m_, and this coincided with the onset of dielectric relaxation at 295 K (Marshall *et al.*, 2020 ▸). Overall, besides its useful dielectric properties, the *x*BZN-(1−*x*)BT system is another potential high-strain piezoceramic material.

The addition of BZN at *x* = 1.0% to the BaTiO_3_ host was observed to stabilize the low-temperature *Amm*2 phase (present in BaTiO_3_ at *T* < 270 K) so that it coexists with the *P*4*mm* phase at 295 K. This indicates that local regions in the BaTiO_3_ host show structural distortion around BZN-rich PNRs, forming local regions of *Amm*2 embedded in the dominant *P*4*mm* superstructure at low (<3.0%) BZN concentrations. For 3.0 < *x* < 4.0%, *Amm*2 becomes the dominant phase at 295 K, indicating that the structural distortion induced by PNRs is sufficiently long range that by *x* = 3.9% the average long-range structure is *Amm*2 throughout the material. This structure corresponds to the peaks in dielectric and piezoelectric coefficients (Paterson *et al.*, 2015 ▸; Wu *et al.*, 2017 ▸; Marshall *et a.*, 2020 ▸). At *x* = 4.0% a discontinuity is observed in that the *Amm*2 diffracted intensity shows a sharp drop alongside an apparent rise in the *P*4*mm* diffracted intensity. The structural changes were correlated by a sharp drop in *d*_33_ and maximum polarization *P*_max_ and the appearance of a trace *Pm* 3 phase at 295 K. Increasing the BZN content from 3.9 to 4.0% appears to result in a sudden loss of long-range ferroelectric order with increasing PNR formation, and it is feasible that this composition marks the limit at which the long-range ferroelectric order imposed by the BaTiO_3_ host starts to break down with increasing BZN addition. Further increases in BZN content cause significant dielectric relaxation and a further decline in *d*_33_, with the piezoelectric limit near *x* = 5.0% at 295 K. Further increases in BZN content up to *x* = 8.0% showed dielectric loops consistent with a lossy relaxor dielectric, indicating some remnant weakly coupled ferroelectric regions which result in considerable energy loss per cycle (Marshall *et al.*, 2020 ▸). At *x* > 8.0%, slimmer dielectric hysteresis loops are present, indicating a transition to a weakly coupled relaxor dielectric where any remnant ferroelectric regions are small and weakly coupled. The resulting low dielectric energy loss on switching is consistent with previous work (Wu *et al.*, 2016 ▸, 2017 ▸; Chen *et al.*, 2015 ▸).

This study evaluates the structure–property relationships between the dielectric and crystalline properties of *x*BZN-(1−*x*)BT in detail in the 3.0 < *x* < 8.0% region using neutron and X-ray data over a wide temperature range. Alongside the composition-driven changes in phase presence in the BZN-BT system observed at 295 K, this work investigates the effect of temperature and composition on phase transitions in the *x*BZN-(1−*x*)BT system and gives insights into how the dielectric properties are coupled to the unit cells and phase transitions within this system.

## 3. Experimental methods   

### 3.1. Powder calcination   

The *x*BZN-(1−*x*)BT precursors were prepared from the end-member precursors. BaTiO_3_ precursor was prepared using BaCO_3_ and TiO_2_ (Sigma–Aldrich 99.95%) by mixing for 24 h in isopropyl alcohol (IPA) using zirconia medium at a 10:1 medium:charge ratio. *x*BZN-(1−*x*)BT precursor powders were mixed by adding stoichiometric quantities of BZN to BaTiO_3_ precursors in initial steps of 1 mol% (metals basis) (Wu *et al.*, 2017 ▸; Othman *et al.*, 2014 ▸) with a measurement uncertainty of ±0.003 g. Prior to mixing, BaCO_3_ and TiO_2_ were dried for 1 h in air at 573 K to remove adsorbed water. The Bi(Zn_2/3_,Nb_1/3_)O_3_ precursor was prepared as for BaTiO_3_ by heating to 573 K prior to mixing. Thermogravimetric analysis/differential thermal analysis was performed to determine suitable ramp rates and dwells for different BZN-BT compositions (Cross, 1987 ▸; Othman *et al.*, 2014 ▸).

The BaTiO_3_ and BZN precursors for sintered powder standards and ceramic samples were made up to 20 g batches to minimize compositional uncertainty. The initial high-resolution X-ray evaluation (Marshall *et al.*, 2020 ▸) and the low-temperature study in this work used archive powder standards, with the maximum uncertainty in composition determined as ±0.051 mol% for compositions 0.0 < *x* < 3.6% and 5.0 < *x* < 10.0%. The maximum uncertainty for compositions 3.6 < *x* < 5.0% was determined to be ±0.033 mol%. Samples for neutron and high-temperature X-ray diffraction used 20 g batches for all compositions 3.0 < *x* < 8.0% with a peak uncertainty of ±0.025 mol%. Compositional uncertainties for the X-ray, neutron and ceramic samples are shown in Table 1 ▸.

The precursors were calcined in closed platinum crucibles within closed alumina crucibles in air for 4 h at maximum dwell temperatures between 1128 and 1218 K. Post-calcining, the powder was weighed to determine the precursor reaction completeness, prior to X-ray characterization. The calcined powder was examined at 295 K using a Bruker D5005 diffractometer with Cu *K*α radiation over a θ–2θ range of 20–90°. The calcined powder was >98% phase pure with small quantities of secondary phases identified mostly as BaTi_2_O_5_ (PDF 04-012-4418; https://www.icdd.com/) and BaTi_5_O_11_ (00-035-0805; O’Bryan *et al.*, 1974 ▸). The precursors were milled with zirconia medium with a 10:1 medium:charge ratio in IPA for a further 20 h to ensure compositional and crystal-size homogeneity prior to sintering. The unit-cell and initial parameters used for phase identification and modelling in the *x*BZN-(1−*x*)BT samples are shown in Table 2 ▸.

### 3.2. Sample preparation for X-ray and neutron diffraction   

Sintered powder standards were prepared from calcined powder precursors by heating in air in closed platinum crucibles between 1463 and 1513 K for a 2 h hot dwell. The quantities for the powder standards were 2–3 g per X-ray standard and 16–18 g for neutron diffraction. Post-sintering, the powder standards were ground in an agate mortar and sieved (60 µm mesh). The weight loss for all compositions was <1%, indicating that no significant mass loss took place from the calcined precursors during sintering.

### 3.3. Ambient and non-ambient X-ray diffraction   

Ambient-temperature high-resolution X-ray measurements were performed using a Phillips Panalytical X’pert diffractometer equipped with a curved Johanson Ge(111) monochromator, giving focused Cu *K*α_1_ radiation. A solid-state Pixcel detector was used with a step size of ∼0.013°, a range of 20–135° 2θ and a total scan time of 15 h at 295 K. Peaks were identified using Panalytical’s *HighScore Plus* software and the ICDD PDF 4+ database. High-temperature X-ray characterization was performed with an Anton Paar furnace stage on the Panalytical diffractometer at 10 K intervals from 300 to 400 K, with a total time of 2.5 h per scan.

Low-temperature X-ray diffraction measurements were performed with a Bruker D5005 with Cu *K*α radiation and an Oxford Instruments Phenix cryogenic He-cooled stage. Measurements were performed down to 20 K for compositions 0.0 < *x* < 20.0%. Low-temperature measurements were performed over a θ–2θ range of 20–90° with a 0.02° step size for a total scan time of 18 h. X-ray measurements provided the baseline structures at 295 K, such that constraints and restraints could be placed on refinements using the lower-resolution data obtained from the Bruker diffractometer [*R*_exp_ = 18.642% for *x* = 3.6% (20 K, Bruker diffractometer) and *R*_exp_ = 4.560% for *x* = 3.6% (295 K, Phillips Panalytical)]. Temperature-driven phase transitions of BaTiO_3_ were used as a control and to enable determination of pure tetragonal, orthorhombic and rhombohedral phases on cooling.

Rietveld refinement for X-ray and neutron data was performed with the *TOPAS-Academic 6* software (Coelho, 2018 ▸) using a Pearson VII model for peak fitting. The best fits were obtained using anisotropic displacement parameters for polar phases and isotropic atomic-dependent parameters for the cubic phase. The use of anisotropic parameters for cubic models led to instabilities during the refinements. Restraints on the lattice parameters were based on those used by Wu *et al.* (2017 ▸) for initial refinements. The tetragonal *x*BZN-(1−*x*)BT phase was fitted using a modified version of the BaTiO_3_ PDF as the baseline (PDF 00-005-0626, *P*4*mm*). The models for orthorhombic *x*BZN-(1−*x*)BT were based on PDF 01-083-8301 for *Amm*2 BaTiO_3_.

Lattice parameters for all the polar phases were allowed to refine within limits determined from the previous study (Wu *et al.*, 2017 ▸). For high-resolution X-ray and neutron data, atoms not on special positions for the polar phases (*B*, O1 and O2 sites) were allowed to refine within ±0.05 of the initial position, with the exception of those near the limits of the unit cell to prevent atom positions <0.0 or >1.0. For impurity phases, lattice parameters were allowed to refine within ±0.05 Å of the initial positions but atoms were fixed. A selection of models were used for refinement to determine the best fit between calculated (*Y*_calc_) and observed (*Y*_obs_) data. Three models were assessed in total, comprising (for *x* < 4.0%) *P*4*mm* + *Amm*2, *P*4*mm* + *Cm* and *P*4*mm* + *Pm* 3 *m*. The quality of the model fits was assessed in terms of minimizing *Y*_calc_ − *Y*_obs_ over the largest possible range of *d* spacing, goodness of fit (GoF, defined as *R*_wp_/*R*_exp_; Young, 1993 ▸), uncertainty in lattice and atomic displacement parameters, and convergence in lattice parameter and atom position trends for the polar phases.

### 3.4. Low-temperature neutron diffraction   

Neutron diffraction data were obtained at ISIS at the STFC Rutherford Appleton Laboratory on the high-resolution powder diffraction (HRPD) beamline, using ∼12 g of powder sample per load. Data from banks 1 and 2 (backscatter and the 90° bank) were used for simultaneous refinement in *TOPAS*. Fitting parameters obtained from the most recent ceria standard (dated 11.12.2019) measured at ISIS were used for the peak-shape functions. Time-of-flight data limits were set between 30 000 and 125 000 µs, equivalent to *d*-spacing limits of 0.65–2.59 Å for a total of 4610 steps per measurement. Compositions *x* = 3.0–8.0% inclusive were evaluated at 290 K by HRPD for 2 h per sample. Compositions *x* = 3.6, 4.0 and 5.0% were evaluated from 20 to 350 K. Counts were taken for 10 min per temperature interval, following a 5 min dwell at 10 K steps, with the exception of *x* = 4.0% where the range 20–120 K was covered in 20 K steps.

### 3.5. Ceramic sample preparation   

Ceramic samples were prepared using the de-agglomerated calcined precursors as described in Section 3.1. Cylindrical pellets of green dimensions 13 mm diameter × 2.7 mm were pressed at 80 MPa with 0.01 ml of 2 wt% aqueous PVA solution as the organic binder. Samples were sintered in a sealed platinum crucible within a powder bed consisting of calcined 0.2BZN-0.8BT to counter Bi_2_O_3_ loss. An inner layer of powder of the same sample composition adjacent to the pellet prevented contamination between the BZN-enriched outer powder bed and the pellets. Pressed pellets were sintered between 1463 and 1513 K for 90–120 min for the hot dwell, then cooled at 0.5 K min^−1^ for 30 K prior to an isothermal hold between 15 and 60 min, and finally cooled at 2 K min^−1^ to room temperature. The sintered density was assessed using the Archimedes method and the theoretical density was calculated from the lattice parameters determined from powder standards. Dense (≥95%) ceramics were polished with silicon carbide paper and sequential diamond suspension to 1 µm roughness and an average thickness of 1 mm. Conductive silver paint electrodes were annealed at 773 K for 1 h prior to measurement to remove stress prior to measurements. Microstructure was examined using scanning electron microscopy (SEM) with a Zeiss SUPRA 55 FEGSEM after thermal etching for 1 h at a temperature 100 K lower than the sintering temperature. To mitigate charging issues, samples were imaged with an accelerating voltage of 5–8 kV and a working distance of 6–8 mm from the pole piece.

### 3.6. Polarization and dielectric measurements   

Dielectric measurements were obtained using a Hewlett Pickard 4192A impedance analyser. Measurements of conductance were obtained between 5 and 56 kHz from ambient temperature up to 523 K. Capacitance and dielectric loss were measured over the frequency range 5–640 kHz up to 650 K. Polarization measurements were performed using an AixACCT TF Analysis A3000 with a TREK high-voltage amplifier at a frequency of 1 Hz at ambient temperature as for previous work (Marshall *et al.*, 2020 ▸).

## 4. Results   

### 4.1. X-ray and neutron data at 295 K   

**Figure 1:**
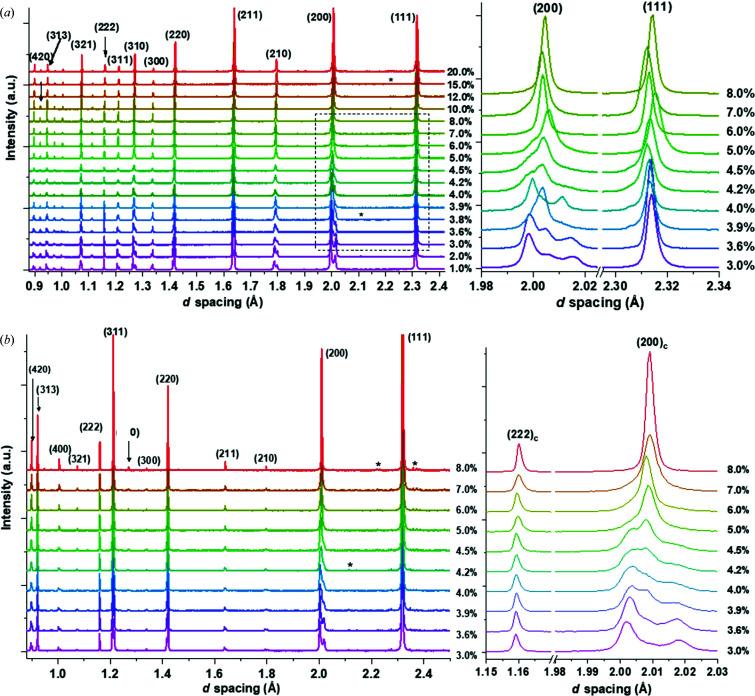
X-ray and neutron data for BZN-BT for (*a*) compositions 1.0 < *x* < 20.0% from X-ray data at 295 K and (*b*) neutron data at 295 K. The enlargements on the right-hand side show the (111)_c_ and (200)_c_ peaks from (*a*) and the (200)_c_ and (222)_c_ peaks from (*b*). Stars mark impurity peaks present post-sintering.

X-ray patterns for 1.0 < *x* < 20.0% and neutron data (bank 1 only) for 3.0 < *x* < 8.0% are shown in Figs. 1 ▸(*a*) and 1 ▸(*b*), respectively. Magnifications of significant pseudocubic peaks for compositions 3.0 < *x* < 8.0% are shown to the right of the main figures.

Some of the differences between the X-ray and neutron data sets, such as the relative intensities of some reflections, are due to the different scattering factors for neutrons and X-rays. Differences in peak shape between the X-ray and neutron data are also present, particularly for compositions 3.6 < *x* < 4.0%. Some of this could be attributable to minor changes in composition between the sintered powder sample batches used for the X-ray and neutron studies, as shown in Table 1 ▸.

The presence of a secondary polar phase was observed from the appearance of a third peak within the (002)/(200)_T_ tetragonal doublet at *x* ≥ 3.0%. However, the high intensity between the (200)/(002)_T_ doublet at 1.0 < *x* < 3.0% indicates either ferroelastic strain or secondary phase presence even at *x* < 3.0%. This peak is observed to increase, reaching a maximum intensity near *x* = 3.9% and then having a rapid decline with a local minimum at *x* = 4.0%. For *x* > 4.0%, the central (200)_pc_ peak increases with subsequent BZN addition and becomes increasingly pseudocubic in character. The (002)/(200)_T_, doublet, after a sharp local maximum at *x* = 4.0%, decreases with increasing BZN content and reaches zero at 295 K near *x* = 7.0% (Marshall *et al.*, 2020 ▸).

### 4.2. Models for diffracted intensity at 295 K   

**Figure 2:**
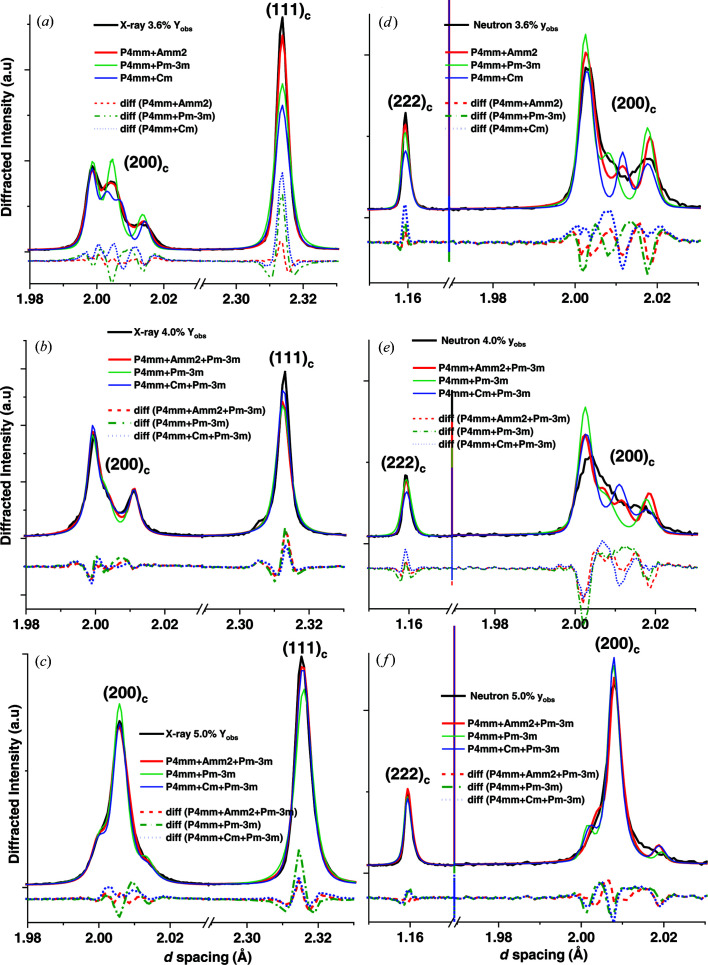
*Y*_obs_, *Y*_calc_ and difference (diff) profiles for the *P*4*mm* + *Amm*2, *P*4*mm* + *Pm* 3 *m* and *P*4*mm* + *Cm* models for compositions *x* = 3.6, 4.0 and 5.0%. (*a*)–(*c*) Model fits from X-ray data at 295 K for (111)_pc_ and (200)_pc_; (*d*)–(*f*) model fits from the neutron data at 290 K for (222)_c_ and (200)_c_.

The accuracies of the Rietveld models were determined from the weighted Bragg factor *R*_wp_, GoF = *R*_wp_/*R*_exp_, and the observed fit between peak positions and models for data acquired at 295 K (Young, 1993 ▸), setting the baseline for non-ambient measurements. Three models were evaluated in *TOPAS* in terms of peak fitting for the X-ray and neutron data: *P*4*mm* + *Amm*2, *P*4*mm* + *Cm* and *P*4*mm* + *Pm* 3 *m*. The *P*4*mm* + *Pm* 3 *m* model acts as a control to monitor the convergence of polar phases to pseudocubic structures. Previous models of this system used *P*4*mm* + *R*3*m* as the structural model for *x* > 3.8% (Wu *et al.*, 2017 ▸) and a rhombohedral phase was identified in an analogous system *x*BZN-(1−*x*)PT (Liu *et al.*, 2018 ▸), but there was little evidence of asymmetry in the 111 peak as would be expected from unit cells with non-90° angles. Rhombohedral peak widths did not account for the breadth of the diffracted intensity not attributed to tetragonal reflections such as (002)/(200)_T_, with significantly poor fits over the entire composition range 1.0 < *x* < 20.0% at 295 K. In addition, the *R*3*m* phase is expected to be present at low temperatures for dilute (*x* < 3.0%) BZN contents, so it would be difficult to explain the presence of two *R*3*m* phases at different temperatures and compositions. A *P*4*mm* + *Cm* model was used since *Cm* is present as a polar phase in other relaxor systems, including NBT-BT (Yao *et al.*, 2012 ▸; Beuerlein *et al.*, 2016 ▸)], alongside tetragonal phases and was shown to be a reasonable fit (Fig. 2 ▸).

Lattice parameters for *P*4*mm* for compositions *x* < 4.0% at 295 K were based on an existing study on Rietveld fitting for the *x*BZN-(1−*x*)BT system (Marshall *et al.*, 2020 ▸). For the *Amm*2 model, the starting lattice parameters were *a*_O_ ≃ *a*_T_, *b*_O_ ≃ *c*_O_ ≃ 2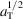. Similarly, the *Cm* model was based on *a*_m_ ≃ *b*_m_ ≃ 2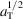, *c*_m_ ≃ *a*_T_, β ≃ 90° and *a*_c_ = (*a*_T_ + *c*_T_)/2 for *Pm* 3 *m* as seen in Table 2 ▸. For *x* > 4.0% all models included *Pm* 3 *m*. Model fits for X-ray and neutron data for compositions *x* = 3.6%, *x* = 4.0% and *x* = 5.0% are compared in Fig. 2 ▸.

Some differences in the peak shape of the 200 triplet peak between the X-ray and neutron data are observed, particularly for *x* = 3.6% [Fig. 1 ▸(*a*)]. Compositional uncertainty is a potential factor in the differences in peak shape, particularly in the range 3.6 < *x <* 4.5%, but the difference in resolution between the neutron and X-ray data is the most probable reason behind the difference in peak shapes. In general, the *Amm*2 + *P*4*mm* models had values of GoF consistently lower than other model fits, as summarized in Table 3 ▸.

The models for the X-ray data show a strong convergence and local minima in GoF near *x* = 4.0%, with little difference in GoF between the *Amm*2-, *Cm*- and *Pm* 3 *m*-based models near *x* = 4.0%. The neutron models from dual-bank refinement showed greater variance in GoF relative to the X-ray models, but both sets of models had a local minimum in GoF at *x* = 3.9% and a global minimum at *x* = 5.0%. The differences in GoF between *Amm*2-, *Cm*- and *Pm* 3 *m*-based models were greater in the neutron models than for X-ray models, but the *Amm*2-based model consistently had the lowest GoF over the full composition range up to *x* = 20.0%. These observations indicate that *Amm*2 is the most probable BZN-stabilized polar phase out of the selected models shown in Fig. 2 and Table 2 ▸.

### 4.3. Diffracted intensity, lattice parameters and atomic displacement at 295 K   

**Figure 3:**
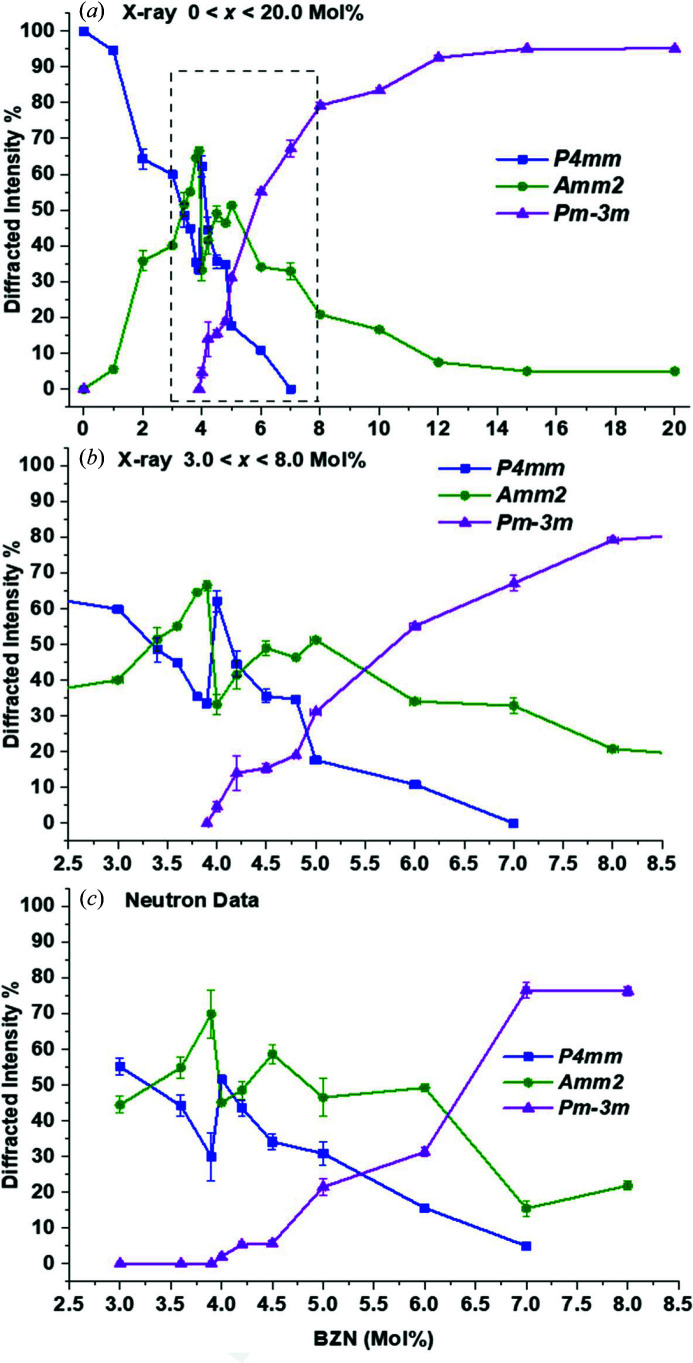
Trends in relative diffracted intensities for the *P*4*mm*, *Amm*2 and *Pm* 3 *m* phases for (*a*) X-ray data for 0.0 < *x* < 20.0% and (*c*) neutron data for 3.0 < *x* < 8.0%. (*b*) A close-up of the region 3.0 < *x* < 8.0% in (*a*).

The X-ray and neutron models enabled the determination of the relative diffracted intensity of different phases in the range 0.0 < *x* < 20.0%. Fig. 3 ▸ displays the relative diffracted intensities attributed to the two polar phases observed in the spectra shown in Fig. 1 ▸. The discontinuity near *x* = 4.0%, corresponding to a sharp spike in tetragonal intensity from both X-ray and neutron data, is consistent with previous examinations of this system (Wu *et al.*, 2017 ▸). Since we are evaluating relative intensity rather than absolute intensity, the spike in *P*4*mm* reflects a sudden drop in *Amm*2 intensity rather than an absolute increase in *P*4*mm*. The discontinuity is at the same composition for both data sets, as is the emergence of cubic phase presence at *T* ≤ 295 K, so it is probable that the bulk of the difference results from instrumental methods rather than compositional differences. The orthorhombic phase is dominant near *x* = 4.0% from both X-ray and neutron data. Both data sets indicate that the tetragonal phase disappears for 6.0 < *x* < 8.0%. From the X-ray data, the ortho­rhombic phase declines in intensity from *x* = 8.0% onwards, reaching a limit of ∼10% of the total diffracted intensity for *x* > 12.0% up to the miscibility limit at 20.0%.

**Figure 4:**
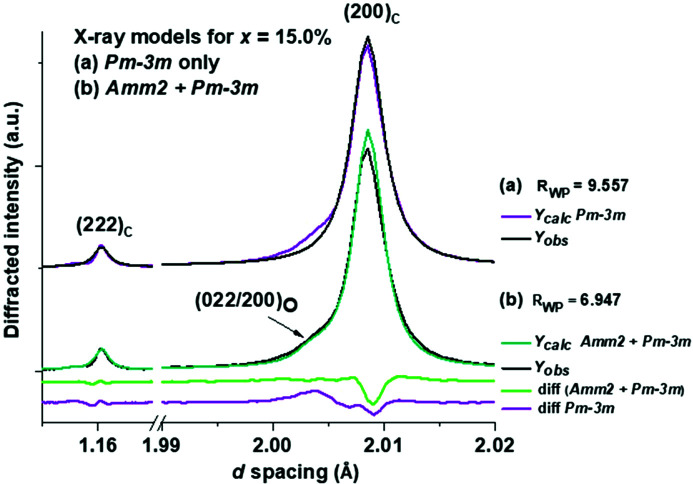
Comparison of (*a*) *Pm* 3 *m*-only and (*b*) *Amm*2 + *Pm* 3 *m* models for *x* = 15.0% and their *R*_wp_ factors. The peak shown in (*b*) is fitted best by the *Amm*2 + *Pm* 3 *m* model.

From Figs. 2 ▸ and 3 ▸, most of the significant phase changes occur in the region 3.0 < *x* < 8.0%. In terms of investigation under non-ambient conditions, we specifically focus on the region 3.6 < *x* < 5.0%, since this is where peak piezoelectric and dielectric coefficients and the discontinuity in *P*4*mm*/*Amm*2 presence occur, and it is of interest to investigate whether any significant temperature-dependent phase transitions occur in this region. The coexistence of *Amm*2 in the paraelectric region, defined as *x* ≥ 5.0%, indicates that the *Amm*2 phase results from the presence of PNRs in terms of a persistent presence of a lower-symmetry phase within a bulk paraelectric matrix up to the miscibility limit near *x* = 20.0%. The persistence of the orthorhombic phase at *x* > 8.0% is observed in the asymmetry of the pseudocubic (200)_c_ when comparing model fits between a *P*4*mm* + *Pm* 3 *m* and a *Pm* 3 *m*-only model at *x* = 15.0% (Fig. 4 ▸).

### 4.4. Crystallography and microstructure   

**Figure 5:**
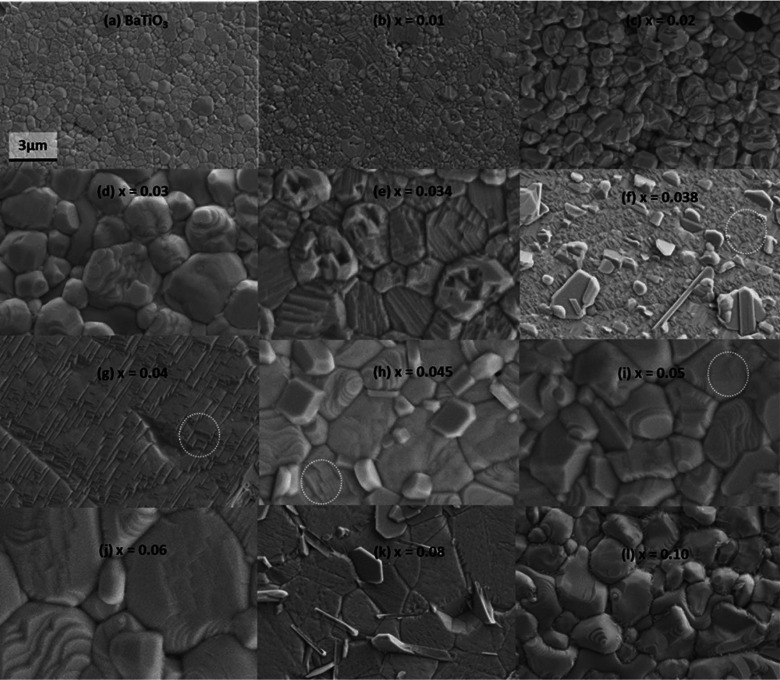
Thermally etched *x*BZN-(1−*x*)BT samples of BZN concentration 0.0 < *x* ≤ 10.0%. All images are at the same equivalent magnification as (*a*). White dotted circles mark the terraced structure most apparent for *x* = 3.8 and 4.0% but also present in compositions where *x* > 4.0%.

The thermally etched, sintered ceramics of compositions 0.0 < *x* < 10.0% were examined by SEM and the resulting images are shown in Fig 5 ▸.

The grain size increased with BZN concentrations up to *x* = 3.8% [Fig. 5 ▸(*f*)], consistent with similar observations from a recent study of this system (Wu *et al.*, 2017 ▸). For compositions *x* > 5.0%, it is unclear how composition affects microstructure since previous studies indicate that compositions 5.0 < *x* < 20.0% have a grain size in the range 1–3 µm (Wu *et al.*, 2016 ▸, 2017 ▸). In this work the BaTiO_3_-like compositions 0.0 < *x* < 3.0% initially had sintered grain sizes of 0.5–1.0 µm, increasing up to 3–4 µm diameter for 3.0 < *x* < 4.5%. The grains also have distinctive terraces, lending credence to the possibility that these terraces are related to ferroelectric domain structure. BaTiO_3_ [Fig. 5 ▸(*a*)] and *x* = 1.0% [Fig. 5 ▸(*b*)] have similar stripe domain patterns, but those for *x* = 2.0% [Fig. 5 ▸(*c*)] and *x* = 3.0% [Fig. 5 ▸(*d*)] show more polygonal terracing. Composition *x* = 3.4% [Fig. 5 ▸(*e*)], with a mixture of striated and polygonal domains, shows a flatter surface than composition *x* = 3.0% [Fig. 5 ▸(*d*)]. The microstructure changes again for compositions *x* = 3.8% [Fig. 5 ▸(*f*)] and *x* = 4.0% [Fig. 5 ▸(*g*)]. These compositions show a flat surface dominated by trigonal terraces, where grain boundaries are infrequent or ill-defined, and correspond to the *Amm*2-dominated compositions at 295 K.

The discontinuity in phase abundance and lattice parameters near *x* = 4.0% is reflected by the reappearance of discrete grains in Fig. 5 ▸(*h*) for *x* = 4.5%. Some grain patterns resembling the terrace structures seen near *x* = 4.0% can be observed for compositions 4.5 < *x* < 6.0%. Another change in grain structure is apparent at *x* = 8.0% [Fig. 5 ▸(*k*)], where the grains have a flatter appearance compared with *x* = 6.0% and *x* = 10.0%. The grain structure for *x* ≥ 10.0% is very similar to that for *x* = 10.0%, with curved irregular grains and fine terraced structures. The crystallographic structure of this material profoundly affects the microstructure and electromechanical properties of the *x*BZN-(1−*x*)BT system.

#### 4.4.1. Lattice parameters at 295 K   

**Figure 6:**
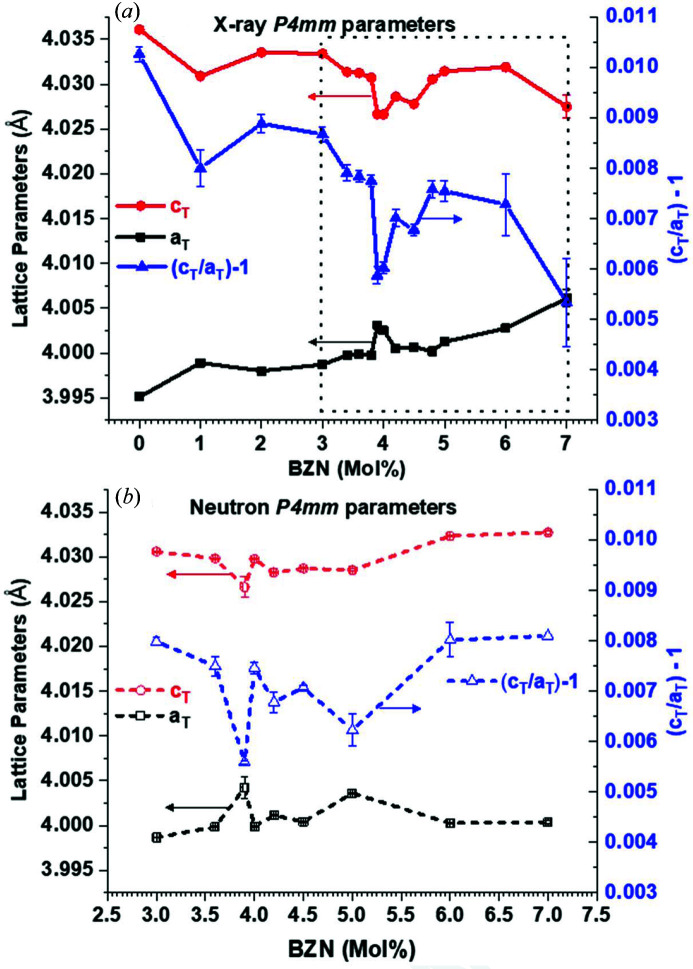
Lattice parameters from *P*4*mm* models for (*a*) X-ray data and (*b*) neutron data alongside the (*c*_T_/*a*_T_) − 1 tetragonal parameter.

The lattice parameters calculated from the *TOPAS* models for the tetragonal and orthorhombic phases are shown in Figs. 6 ▸ and 7 ▸, respectively.

The tetragonal lattice parameters from the X-ray and neutron data show similar trends, namely a slow convergence in *a*_T_ and *c*_T_ with increasing BZN content, then sharp local minima in (*c*_T_/*a*_T_) − 1 near *x* = 4.0%. The actual point of convergence differs slightly between data sets but is in the range 3.9 < *x* < 4.0%, as shown in Fig. 6 ▸.

**Figure 7:**
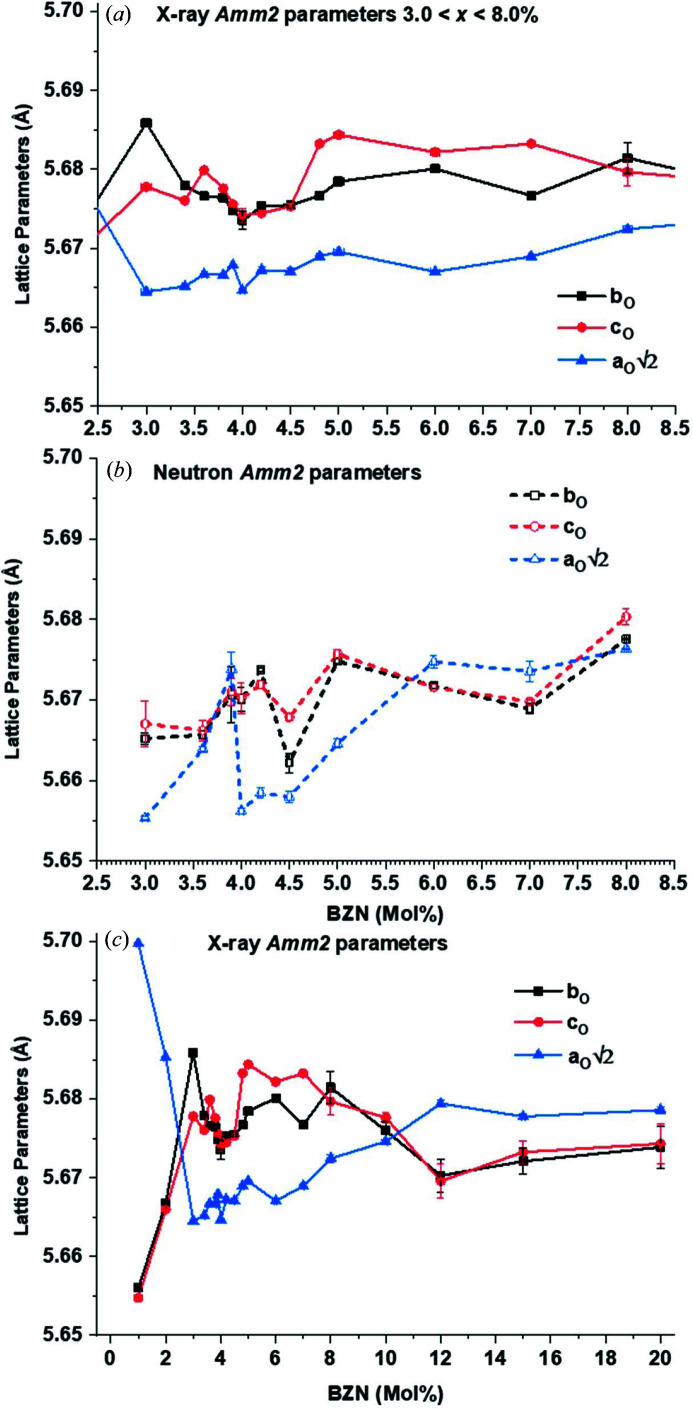
Orthorhombic parameters from X-ray and neutron data at 295 K. (*a*) X-ray data for 3.0 < *x* < 8.0%, (*b*) neutron data for 3.0 < *x* < 8.0% and (*c*) X-ray data for 1.0 < *x* < 20.0%, where *x* = 1.0% is the lower limit for diffracted intensity attributed to *Amm*2 at 295 K.

The orthorhombic parameter trends show that 2^1/2^ *a*_O_ is generally less than *b*_O_ (≃ *c*_O_) over the composition range 3.0 < *x* < 5.0% from the X-ray data [Figs. 7 ▸(*a*) and 7 ▸(*c*)]. There is a convergence between 2^1/2^ *a*_O_ and *b*_O_, *c*_O_ in the range 3.8 < *x* < 4.0%; the values of *b*_O_ are also closest to *c*_O_ in this composition range, with 2^1/2^ *a*_O_, *b*_O_ and *c*_O_ within 0.01 Å. The neutron data in Fig. 6 ▸(*b*) and 7(*b*) show similar trends of 2^1/2^ *a*_O_ < *b*_O_, *c*_O_ for 3.0% to the X-ray data, except for the region 3.6 < *x* < 4.0% where 2^1/2^ *a*_O_ > *b*_O_, *c*_O_ and has a local maximum at *x* = 3.9%. The lattice parameters 2^1/2^ *a*_O_, *b*_O_ and *c*_O_ calculated from neutron data at *x* = 3.9% are all within 0.005 Å, showing a strong convergence to a pseudocubic structure at *x* > 3.9%. Both data sets indicate that, within the uncertainties, 2^1/2^ *a*_O_ < *b*_O_, *c*_O_ for 1.0 < *x* < 8.0%, with the neutron data showing a strong convergence in *Amm*2 parameters at 3.9 < *x* < 4.0%.

#### 4.4.2. Atom site parameters for *P*4*mm* at 295 K   

**Figure 8:**
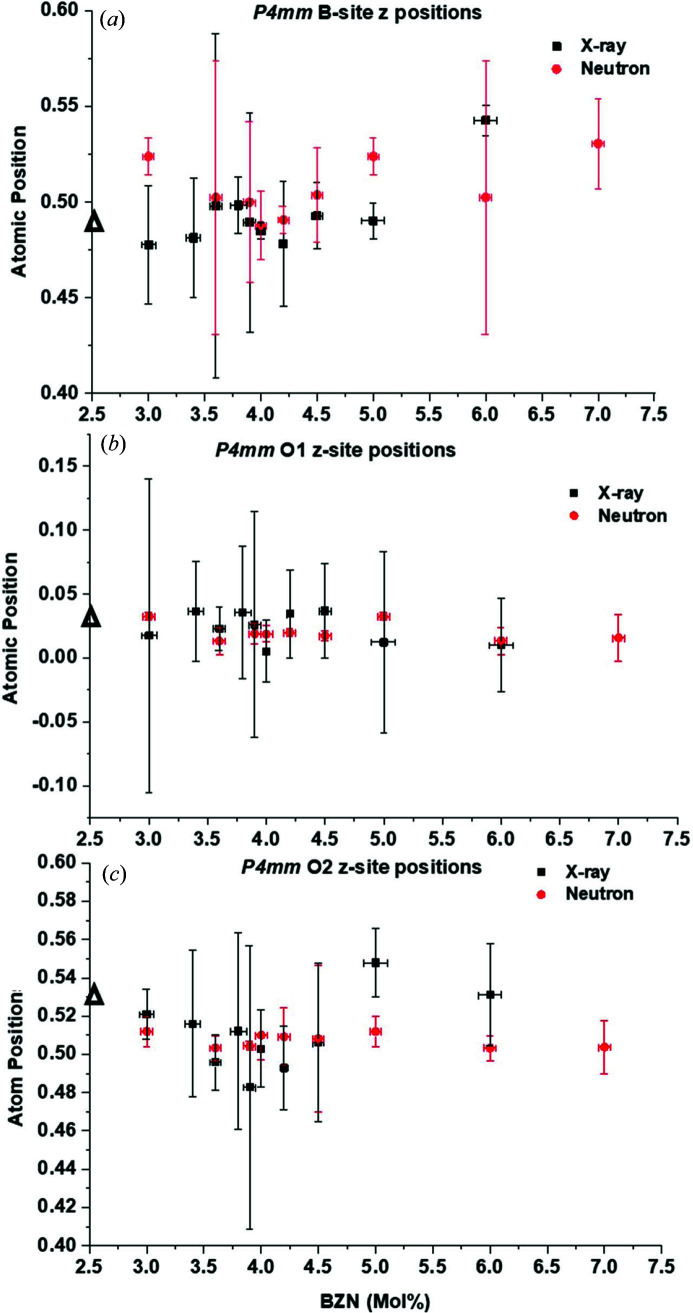
Atomic positions with BZN concentration from *P*4*mm* models. (*a*) *B*-site atom *z* position, (*b*) O1-site atom *z*-position and (*c*) O2-site *z* position. Black triangles show the atom position from X-ray models for BaTiO_3_ at 295 K.

Both *P*4*mm* and *Amm*2 models show a strong convergence to a more cubic appearance over the narrow composition range 3.9 < *x* < 4.0%. The lattice parameters are dependent on atomic positions, specifically the *B*-site atoms and oxygen positions for the *P*4*mm* and *Amm*2 unit cells. The atomic displacements from the *z* positions of the *B* site and the two oxygen sites over the compositions 3.0 < *x* < 8.0% for the *P*4*mm* models are shown in Fig. 8 ▸. Black triangles denote the placements for BaTiO_3_ at 295 K from X-ray data since no neutron data are available for BaTiO_3_ from this work.

For the X-ray data, the *B*-site *z* value has a maximum of *z* = 0.596 ± 0.015 at *x* = 1.0% [not shown in Fig. 8 ▸(*a*)] then decreases to near *z* = 0.48, remaining constant within the uncertainty for 3.0 < *x* < 3.6% near *z* = 0.475 ± 0.03 until an increase at *x* = 3.8% to *z* = 0.498 ± 0.035. Subsequent BZN addition caused a slow decrease in the *B*-site *z* value up to *x* = 4.5%. For *x* > 4.5%, *z* increases, reaching a maximum of *z* = 0.543 ± 0.008 at *x* = 6.0%, the effective tetragonal limit for quantitative X-ray data. Conversely, the neutron data showed a local maximum in *B*-site *z* value of *z* = 0.523 ± 0.01 at *x* = 3.0%, with a decrease in *z* position to a local minimum at *x* = 3.9% of *z* = 0.500 ± 0.042 although the uncertainty in position is very high for the neutron data at *x* = 3.9%. The discontinuity in *B*-site position at *x =* 4.0% where *z* = 0.488 ± 0.018 is similar to that for the X-ray data. The *B*-site *z* positions for neutron data follow similar trends to the X-ray data for 4.0 < *x* < 6.0%.

The trends in O1-site position with BZN addition are less obvious for both data sets. The X-ray data show a relatively flat trend with BZN addition for *x *> 1.0%. For *x* = 1.0%, *z* rises to 0.098 ± 0.005 from *z* = 0.035 ± 0.06 for BaTiO_3_. There is then a flat trend with increasing BZN, as seen in Fig. 8 ▸(*b*), with the *z* position increasing to *z* = 0.036 ± 0.052 at *x* = 3.8%. A local minimum at *x* = 4.0% of *z* = 0.005 ± 0.024 is observed for the O1 site, which is evident even when the uncertainty is taken into account. Conversely, the neutron data do not show any significant composition-driven change in O1 position until a local maximum at *x* = 5.0% of *z* = 0.033 ± 0.003, near the limit of ferroelectricity at 295 K. The O2 positions in *P*4*mm* show similar trends in that the *z* position decreases with BZN addition with a local minimum at *x* = 3.9% for both X-ray and neutron data. The trends in O2 *z* position with composition are more marked for X-ray data than for neutron data, but the lower uncertainty for neutron data (*z* = 0.483 ± 0.074 and *z* = 0.504 ± 0.002 at *x* = 3.9% for X-ray and neutron data, respectively) indicates that the composition trends for the *P*4*mm* O2-site positions are real but relatively minor, relative to the *B*-site *z* positions. The uncertainty in atom positions is generally highest for compositions near *x* = 4.0%, with the uncertainty in atom positions generally less for neutron data than for X-ray data, particularly for oxygen data.

#### 4.4.3. Atom site parameters for *Amm*2 at 295 K   

**Figure 9:**
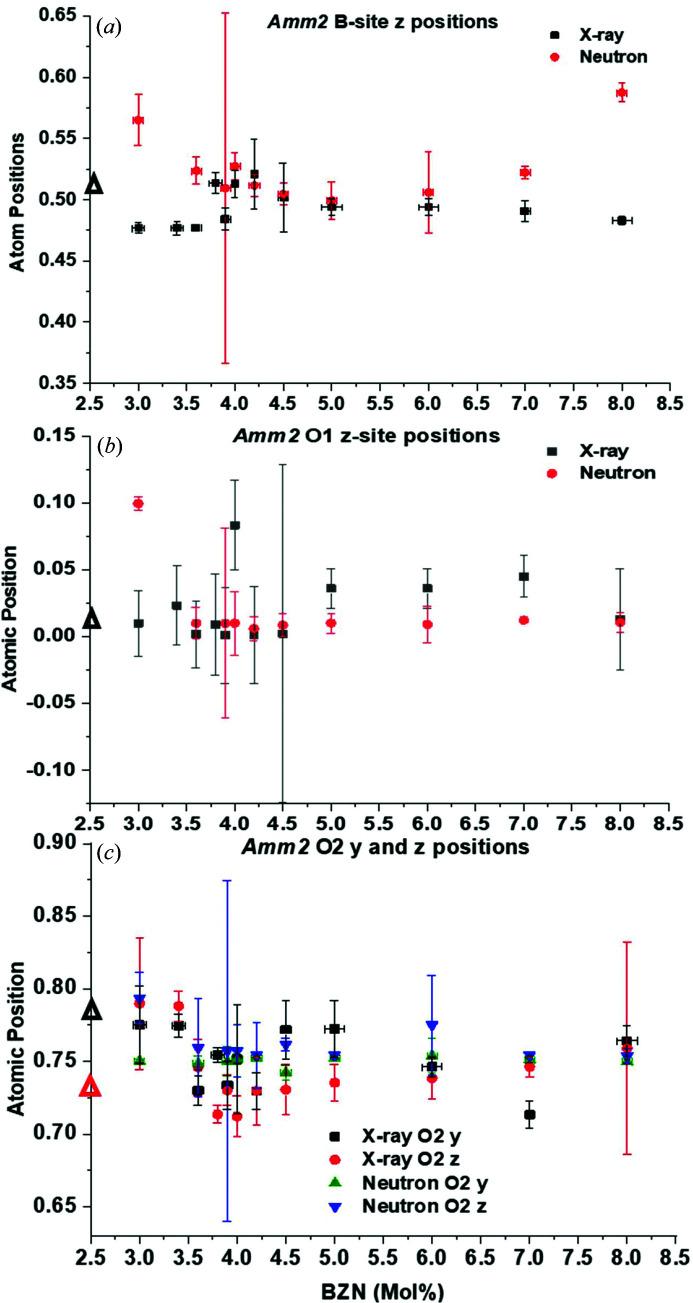
Atomic positions with BZN concentration for *Amm*2 models. (*a*) *B*-site atom *z* position, (*b*) O1-site atom *z* position and (*c*) O2-site *z* position. Black triangles show the atom position from X-ray models for *x* = 1.0% at 295 K for (*a*) and (*b*). For (*c*), black and red triangles are for the *y* and *z* positions at *x* = 1.0%, respectively.

Composition-driven trends in *B*-site, O1-atom and O2-atom positions for unit cells from the *Amm*2 models are shown in Fig. 9 ▸.

The trends in *B*-site *z* positions for *Amm*2 are less obvious when considering the X-ray and neutron data together, as seen in Fig. 9 ▸(*a*). The neutron data indicate an initial increase in *B*-site *z* position with BZN addition. The X-ray data show a decrease, relative to *x* = 1.0%, the most dilute BZN concentration, where *Amm*2 coexists with *P*4*mm* at 295 K. After the initial decrease from *z* = 0.504 ± 0.012 for *x* = 1.0%, the *B*-site *z* position increases to *z* = 0.514 ± 0.009 at *x* = 3.8%, a local maximum. This is then succeeded by a sudden drop at *x* = 3.9% where *z* = 0.484 ± 0.009, before the value returns to *z* = 0.513 ± 0.011 at *x* = 4.0%. The X-ray and neutron data do show some convergence over 3.6 < *x* < 5.0%, with the neutron data exhibiting a similar dip at *x* = 3.9% to the X-ray data, except that the uncertainty for the neutron data is very high for the *Amm*2 model. This is a general feature for the neutron data from the *Amm*2 model for *x* = 3.9%, as seen in Fig. 9 ▸. Over the region 4.0 < *x* < 5.0%, the *B*-site *z* positions from X-ray and neutron data show a true convergence, coinciding with the ferroelectric-to-relaxor transition regime as seen in previous work (Marshall *et al.*, 2020 ▸). The local minima in *z*-position values are metastable at around *z* = 0.499 ± 0.01 at *x* = 5.0, the effective ferroelectric limit at 295 K.

The O1-site *z* positions for *Amm*2 in Fig. 9 ▸(*b*) show a more disordered response with BZN addition from X-ray and neutron data. The neutron data show a maximum in O1-site *z* position at *x* = 3.0% with *z* = 0.10 ± 0.005, which decreases to an equilibrium value near *z* = 0.01 ± 0.01 for subsequent BZN addition. In contrast, the X-ray data show a more erratic trend with BZN, with similar uncertainties to neutron data and with an absolute maximum at *x* = 4.0% of *z* = 0.084 ± 0.036. Both data sets converge over 4.0 < *x* < 5.0% as for the *B*-site *z* positions, where *x* = 4.5% marks a convergence for data sets near *z* = 0 although the uncertainty is very high. The O2 *Amm*2 position has degrees of freedom in both *y* and *z*, as shown in Fig. 9 ▸(*c*). Both X-ray and neutron data show similar trends to the neutron data *B*-site positions with increasing BZN addition, although the *z* position has more of an increase than the *y* position for 1.0 < *x* < 3.0%. Both *y* and *z* positions show local minima at *x* = 3.9% with an average value for *z* = 0.74, again with a high uncertainty for the neutron *z* position. Up to *x* = 6.0, the average X-ray values for the O2 positions are *y* ≃ 0.77 and *z* ≃ 0.73, respectively, with the neutron data showing *y* ≃ *z* ≃ 0.755. The high uncertainty in the neutron data in the 3.9 < *x* < 4.5% region is indicative of a highly deformed unit cell where atom positions can have a wide range of displacements, particularly at *x* = 3.9%.

## 5. Summary of dielectric measurements   

### 5.1. Polarization and dielectric measurements   

**Figure 10:**
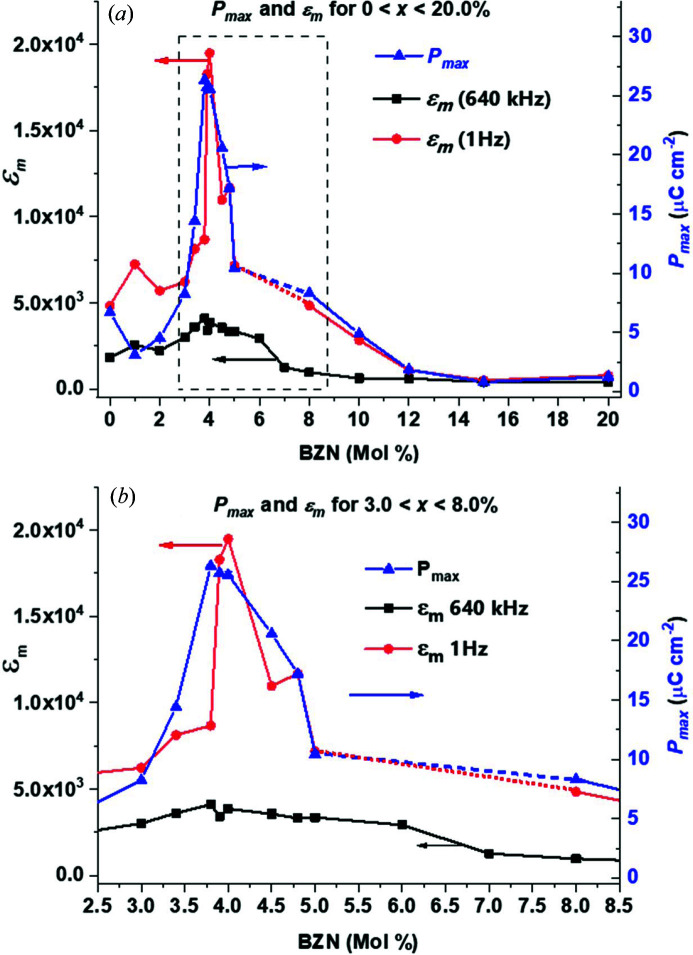
Trends in *P*_max_, ɛ_m_ (640 kHz) and ɛ_m_ (1 Hz) with BZN addition over (*a*) 0.0 < *x* < 20.0% and (*b*) 3.0 < *x* < 8.0%. For compositions *x* = 6.0 and 7.0%, data could not be obtained at 1 Hz owing to the high conductivity of the samples.

Measurements of the dielectric properties of the *x*BZN-(1−*x*)BT system have been reported in detail in previous work (Wu *et al.*, 2016 ▸, 2017 ▸; Paterson *et al.*, 2015 ▸; Marshall *et al.*, 2020 ▸). The general consensus is that for small BZN concentrations (1.0 < *x* < 3.0%) the dielectric constant rises with increasing BZN addition, with a dielectric maximum in the region of 3000 < *ɛ*_m_ < 4000 (640 kHz) in the range 3.8 < *x* < 5.0% (Wu *et al.*, 2017 ▸; Marshall *et al.*, 2020 ▸). The trends in the dielectric constant *ɛ*_m_ at 640 kHz and 1 Hz and the maximum polarization *P*_max_ are shown in Fig. 10 ▸.

The initial BZN additions cause a drop in *P*_max_ at *x* = 1.0%, relative to BaTiO_3_, with *ɛ*_m_ showing a slow increase up to *x* = 3.0%. For 3.0 < *x* < 4.0%, *P*_max_ and *ɛ*_m_ increase significantly, all having maxima in the range 3.8 < *x* < 4.0%. The maximum values for *ɛ*_m_ were 19 487 at *x* = 4.0% and 4098 at *x* = 3.8% for 1 Hz and 640 kHz, respectively, with *P*_max_ = 26.3 µC cm^−2^ at *x* = 3.8%. The values of *ɛ*_m_ and *P*_max_ remain high up to *x* = 4.0%.

Further BZN additions caused a sharp drop in *P*_max_, and more irregular, lossy polarization loops, with *x* = 5.0% showing the characteristics of a relaxor ferroelectric (Marshall *et al.*, 2020 ▸). The drop in polarization is mirrored by a similar drop in *ɛ*_m_ at 1 Hz for *x* > 4.0%. The dielectric permittivity at 640 kHz does not significantly decrease for *x* > 4.0% but remains >3000 up to *x* = 6.0%, with *ɛ*_m_ at 1 Hz and 640 kHz converging for 12.0 < *x* < 15.0%. Comparing *ɛ*_m_ values for 1 Hz and 640 kHz indicates some degree of dispersion present at any BZN concentration.

A similar decline in *d*_33_ with BZN addition was observed by Marshall *et al.* (2020 ▸), from a maximum *d*_33_ = 110 pC N^−1^ at *x* = 3.9% to *d*_33_ = 10 pC N^−1^ at *x* = 4.2%. The trends in piezoelectric and dielectric coefficients with BZN content, particularly in the range 3.6 < *x* < 5.0%, reflect those seen with the drop in *Amm*2 intensity at *x* ≥ 4.0%. The trends in *ɛ*_m_ at low frequencies are strongly influenced by ferroelectric order, as seen by the sudden decrease in magnitude alongside *P*_max_ at *x* > 4.0%, coinciding with the lattice parameter convergence and distortion of atom displacement seen in Figs. 6 ▸–9 ▸ ▸ ▸.

## 6. Non-ambient diffraction data   

### 6.1. Low-temperature X-ray diffraction   

**Figure 11:**
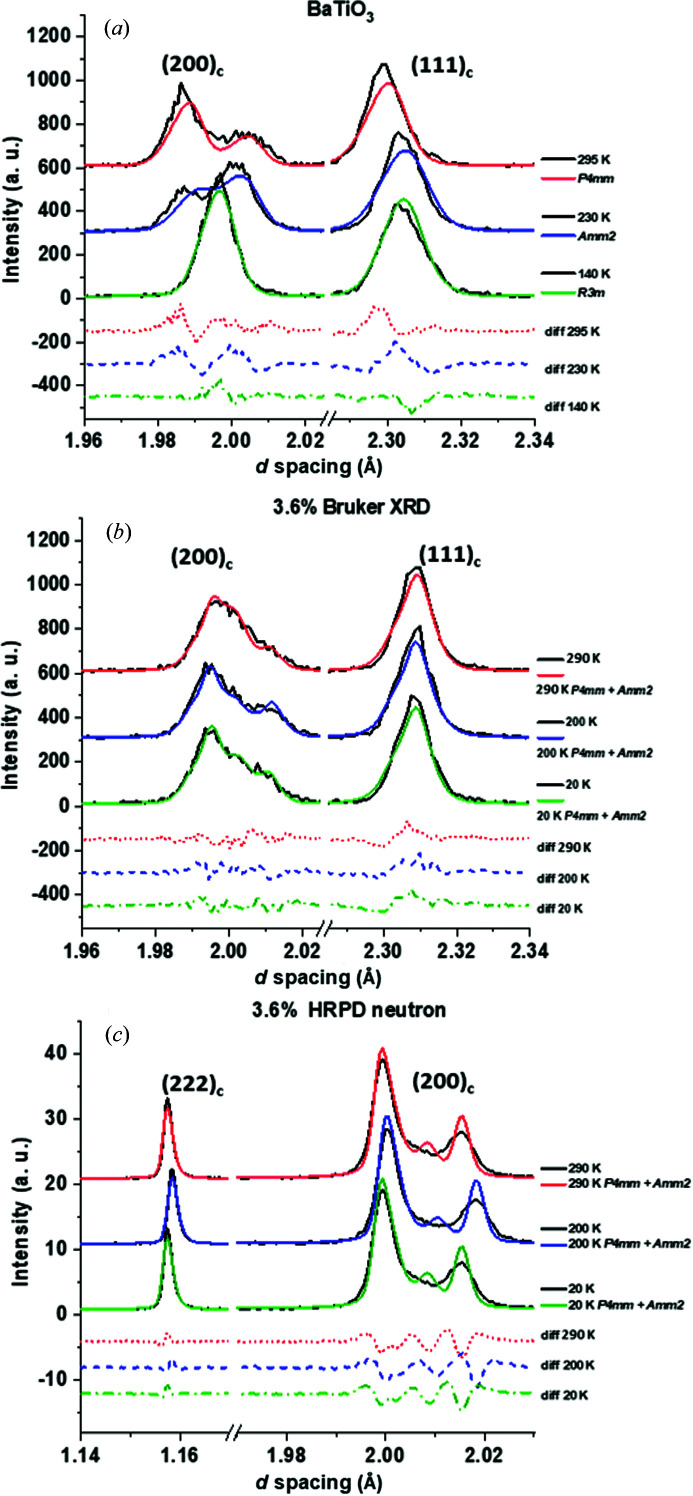
Low-temperature diffraction data and models for (*a*) BaTiO_3_ (X-ray), (*b*) *x* = 3.6% (X-ray) and (*c*) *x* = 3.6% (neutron). BaTiO_3_ data were obtained at 295, 230 and 140 K; data from composition *x* = 3.6% were obtained at 290, 200 and 20 K.

Crystallographic data from X-ray and neutron scattering were obtained over the temperature range 10–400 K. The values of *R*_exp_ from the X-ray models were ∼18.5% from low-temperature Bruker data and ∼10.5% from high-temperature data. The models for the low-temperature neutron data had *R*_exp_ ≃ 3.3%. Model fits and data for the low-temperature X-ray BaTiO_3_ and *x* = 3.6% data are shown alongside neutron data and models for *x* = 3.6% in Fig. 11 ▸.

For BaTiO_3_, the change in peak intensities in the (002)/(200)_T_ doublet for *P*4*mm* to (022)/(200)_O_ for *Amm*2 is seen between 295 and 230 K. The single (202)_R_ reflection and the broadening of the (111)_c_ peak to the (201)/(003)_R_ doublet indicates an *R*3*m* unit cell. Any unit cell with non-90° angles would have a broader asymmetric (111)_c_ peak relative to a unit cell where α = β = γ = 90°, hence the use of BaTiO_3_ as a unit-cell standard. All models had some difficulty providing an accurate fit from the neutron data for the (200)_c_ doublet, irrespective of composition, as seen in Fig. 11 ▸(*c*).

### 6.2. Non-ambient diffraction data   

**Figure 12:**
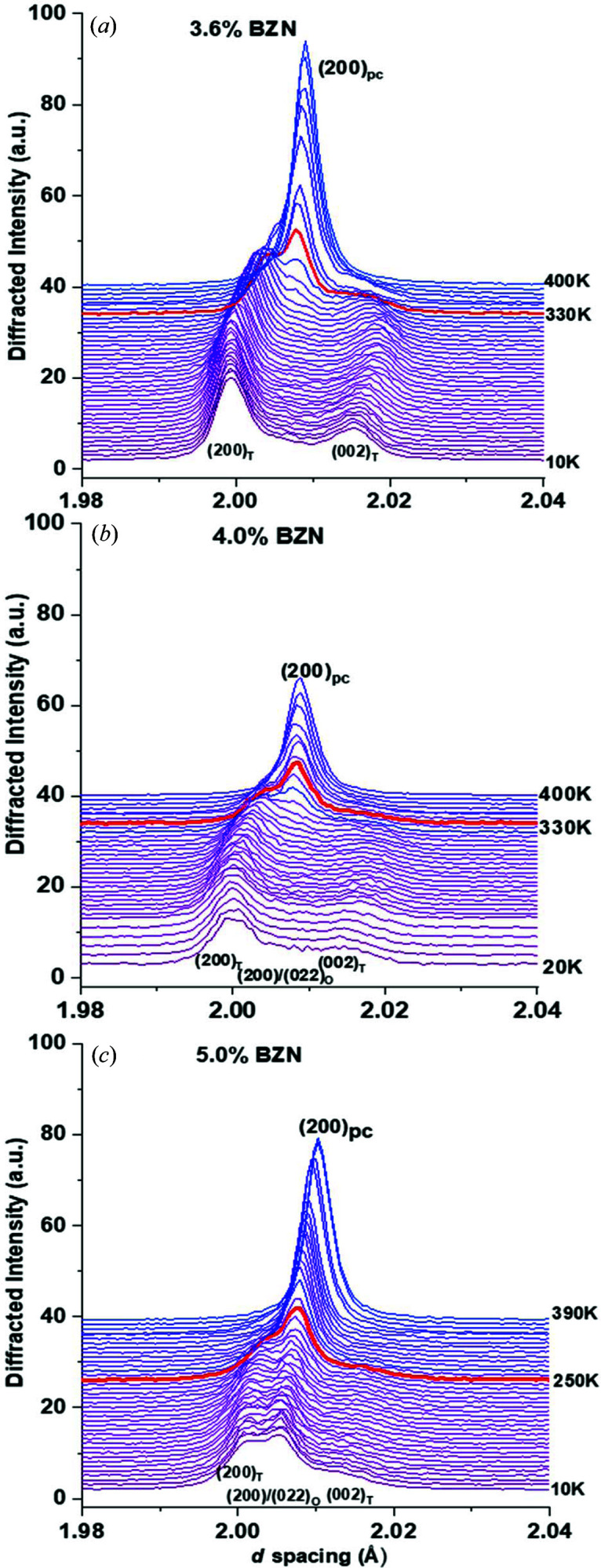
Combined neutron (*T* < 350 K) and high-temperature X-ray (350 < *T* ≤ 400 K) tomographs for (*a*) *x* = 3.6%, (*b*) *x* = 4.0% and (*c*) *x* = 5.0%. The plot corresponding to the known *T*_m_ is highlighted in red. *T*_m_ for 5.0% was estimated following Wu *et al.* (2017 ▸).

Combined high-temperature X-ray and neutron data tomographs of compositions *x* = 3.6, 4.0 and 5.0% are shown in Fig. 12 ▸. The data are coincident with known values of *T*_m_ (640 kHz) for *x* = 3.6 and 4.0%, and the value of *T*_m_ estimated by Wu *et al.* (2017 ▸) for *x* = 5.0%

All three compositions in Fig. 12 ▸ show an increase in the *c*_T_/*a*_T_ ratio with decreasing temperature, with maxima in *c*_T_/*a*_T_ of around 150 < *T* < 200 K as observed by the separation of the (002)/(200)_T_ doublet. For *T* < 200 K a more orthorhombic character is apparent, as seen from the increase in magnitude of the (022)(200)_O_ peak in composition *x* = 5.0%, but this trend is much reduced for compositions *x* = 3.6 and 4.0%, which show a more tetragonal character with decreasing temperature.

**Figure 13:**
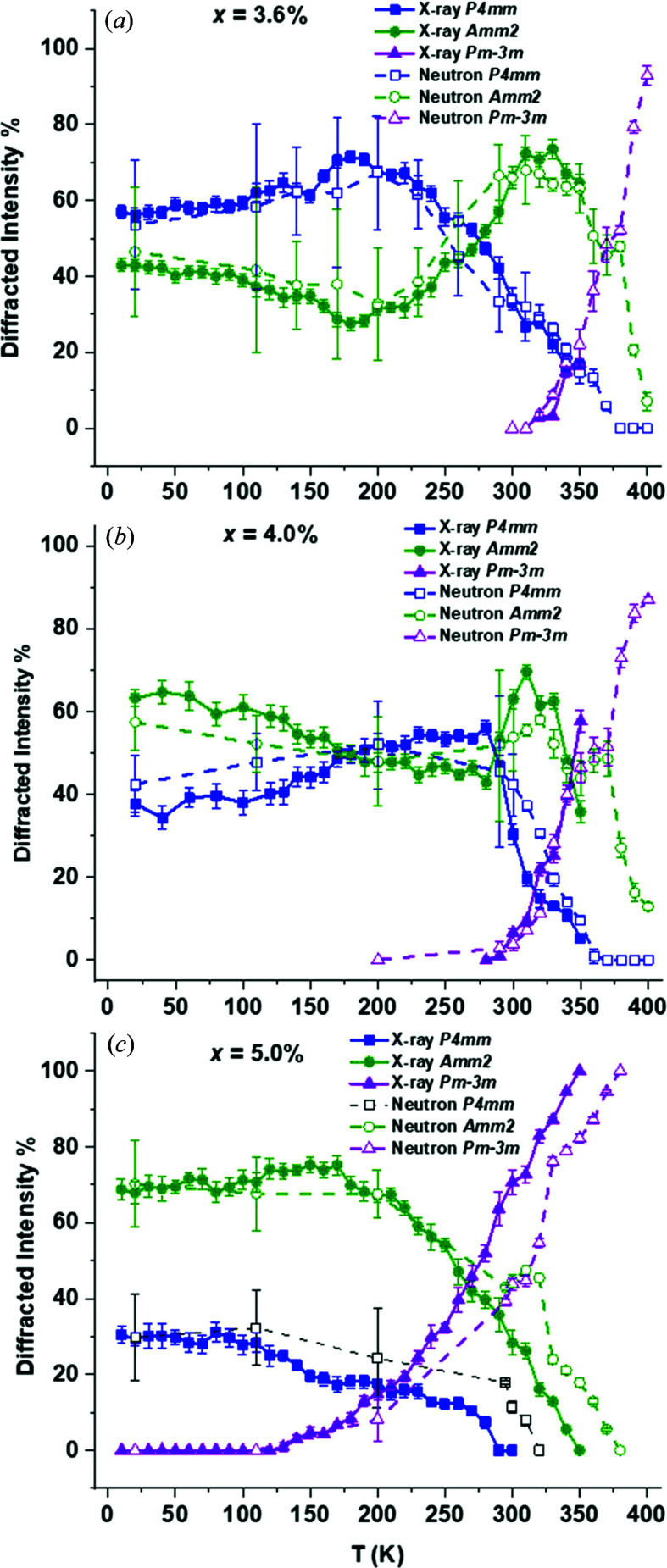
Trends in phase abundance with temperature from combined non-ambient X-ray and neutron data for (*a*) 3.6%, (*b*) 4.0% and (*c*) 5.0%. Hollow points and dashed lines are neutron data; solid points and lines are X-ray data.

The peak-fitting models also showed a poorer GoF for all compositions in the region 150 < *T* < 220 K, with the poorest fits for composition *x =* 4.0%. The central (200)_c_ peak at 400 K for *x* = 4.0% is lower than those for *x* = 3.6% and *x* = 5.0%, indicating that composition 4.0% still has some orthorhombic character at 400 K and is not fully paraelectric at *T* = 400 K. In general, the model fits are improved for structures dominated by a single phase such as 3.6% at 200 K and 5.0% at 290 K, relative to those with mixed polar phase presence. Trends in phase abundance attributed to *P*4*mm*, *Amm*2 and *Pm* 3 *m* from X-ray and neutron models are shown in Fig. 13 ▸.

### 6.3. Temperature dependence of phase presence for 3.6 < *x* < 5.0%   

Lattice parameters were derived from the dual-bank neutron data obtained from compositions *x* = 3.6, 4.0 and 5.0%. Refinements in *TOPAS* enabled the determination of lattice parameters alongside a tentative determination of zero-frequency *T*_m_ for composition 5.0%. The values of *T*_m_ for 5 kHz and 640 kHz from previous work (Wu *et al.*, 2017 ▸; Marshall *et al.*, 2020 ▸) are shown in Table 4 ▸.

**Figure 14:**
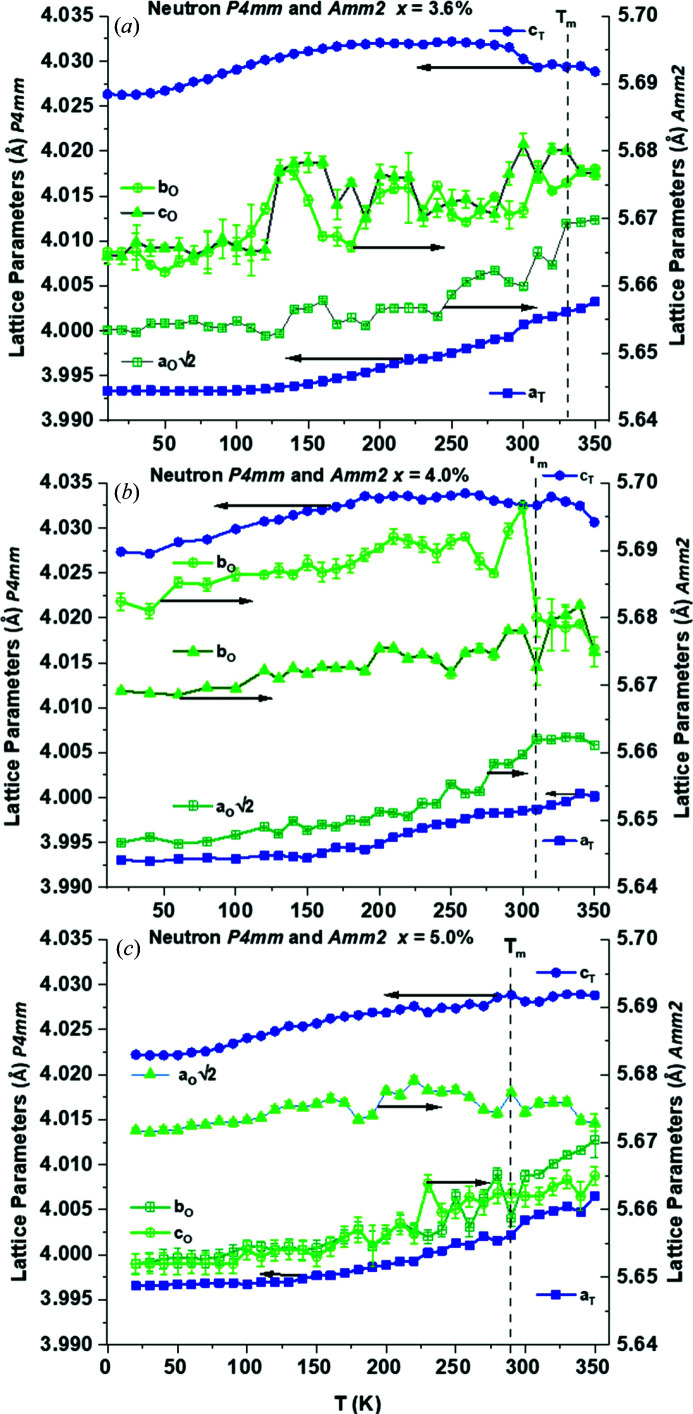
Temperature dependence of lattice parameters for *P*4*mm* and *Amm*2 phases for (*a*) 3.6%, (*b*) 4.0% and (*c*) 5.0%. *T*_m_ (640 kHz) as determined from previous studies (Wu *et al.*, 2017 ▸; Marshall *et al.*, 2020 ▸) is shown by the dashed line.

When Table 4 ▸ is compared with the trends in diffracted intensity in Fig. 13 ▸ for *x* = 3.6% and *x* = 4.0%, *T*_m_ is observed to coincide with the point where the intensities attributed to *P*4*mm* and *Pm* 3 *m* are equal for both X-ray and neutron data. For *x* = 5.0%, there is a gap between the X-ray and neutron data, but this is most likely a result of the poor resolution of the low-temperature X-ray data. The temperature at the midpoint of the X-ray and neutron data where the *P*4*mm* and *Pm* 3 *m* intensities cross is ∼250 K. From the frequency dependence of *T*_m_ at *x* = 5.0% from prior work (Paterson *et al.*, 2015 ▸; Wu *et al.*, 2016 ▸, 2017 ▸; Chen *et al.*, 2015 ▸; Marshall *et al.*, 2020 ▸) and Fig. 10 ▸, 250 K is estimated to be close to the zero-frequency value of *T*_m_ at *x* = 5.0%. There are changes in phase abundance with temperature as shown in Figs. 12 ▸ and 13 ▸, but it is evident that, for *x* ≥ 3.6%, there are no significant temperature-driven phase transitions in the *x*BZN-(1−*x*)BT system. This is also reflected in the trends in lattice parameters for the polar *P*4*mm* and *Amm*2 in Fig. 14 ▸.

The *P*4*mm* and *Amm*2 models show lattice parameter contraction with decreasing temperature. For the pseudo-classical ferroelectric composition *x* = 3.6% the tetragonal parameters show some expansion around 150 < *T* < 200 K. The *Amm*2 parameters show an apparent expansion around 150 K, but it is less well defined than that for *P*4*mm*. Lattice parameter convergence for *Amm*2 is observed near *T*_m_, but it is less well defined for *P*4*mm* and takes place over a range of ∼30 K starting at 300 K, 20 K lower than *T*_m_ at *x* = 3.6%. Lattice parameter convergence for *Amm*2 was most marked for *x* = 4.0%, which coincided with *T*_m_, with a minor decrease in *c*_T_ at *T*_m_. No defined contraction in lattice parameters was observed for *x* = 5.0%, but a general convergence for *P*4*mm* and *Amm*2 from *T* = 270 K is apparent.

#### 6.3.1. Temperature-dependent phase transitions   

**Figure 15:**
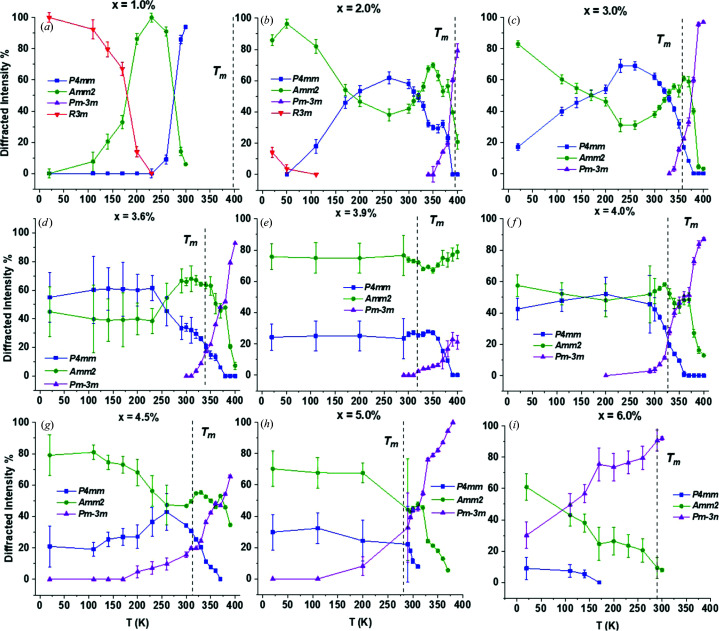
Phase abundance with temperature for X-ray data for (*a*) *x* = 1.0%, (*b*) *x* = 2.0%, (*c*) *x* = 3.0%, (*d*) *x* = 3.6%, (*e*) *x* = 3.9%, (*f*) *x* = 4.0%, (*g*) *x* = 4.5%, (*h*) *x* = 5.0% and (*i*) *x* = 6.0%. Dashed lines indicate *T*_m_ (5 kHz) for the specific composition.

The low resolution from the Bruker X-ray data is not sufficient for lattice-parameter determination but gives a qualitative overview of phase abundance even when the uncertainty is taken into account. Plots of phase abundance with temperature for all non-ambient X-ray data over compositions 1.0 < *x* < 6.0% are shown in Fig. 15 ▸.

The trends in phase abundance with temperature from X-ray data were compatible with the low-temperature neutron data shown alongside the X-ray data in Fig. 13 ▸ for compositions *x* = 3.6, 4.0 and 5.0%. Similarly, the higher-resolution high-temperature X-ray data are also consistent with neutron data for 290 < *T* < 350 K. Some differences between the X-ray and neutron data are apparent, particularly for composition *x* = 3.6%, but this is most likely an artefact of the different resolution between data sets rather than systematic error. Similar differences between X-ray and neutron data exist for composition *x* = 5.0% with respect to the increase in cubic intensity with temperature in Fig. 15 ▸(*h*). For composition *x* = 3.6% from the ferroelectric regime, the tetragonal intensity drops before the first appearance of the cubic phase, resulting in a high-temperature orthorhombic dominated phase around *T*_m_. Something similar was seen for *x* = 4.0% over a narrower temperature range. For *x* = 5.0%, no well defined ortho­rhombic region is evident so it is possible that a high-temperature orthorhombic phase is specific to ferroelectric compositions where *x* < 5.0%.

### 6.4. Phase diagram of *x*BZN-(1−*x*)BT for 0.0 < *x* < 20.0%   

**Figure 16:**
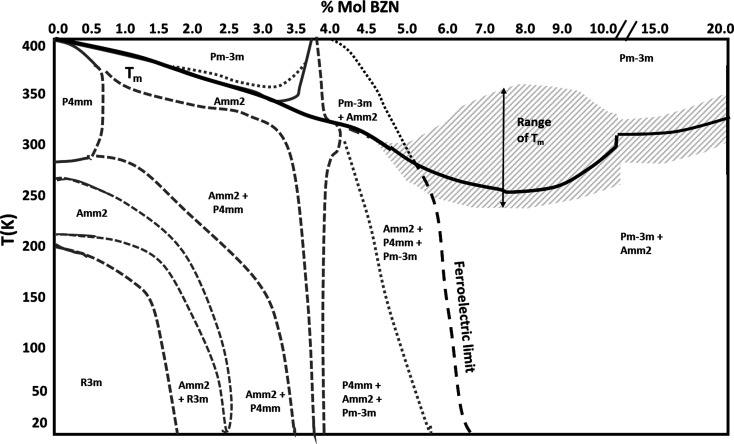
Low-temperature phase diagram of the *x*BZN-(1−*x*)BT system for 0.0 < *x* < 20.0%. The ferroelectric limit is at or near 5.0% at 295 K. The break in the *x* axis marks the transition from lossy relaxor to low-loss relaxor at *x* ≥ 15.0%. Values of *T* for compositions 5.0 < *x* < 15.0% are based on prior studies (Wu *et al.*, 2016 ▸, 2017 ▸).

From Fig. 15 ▸, the low-temperature X-ray data from this study are sufficient for predicting trends in phase transitions and dominance with temperature in line with the neutron data, alongside the values of *T*_m_ in Table 4 ▸. By collating the data shown in Fig. 13 ▸, Table 4 ▸ and Fig. 15 ▸, we can determine the temperature–composition phase diagram from 20 to 400 K and for 0.0 < *x* < 20.0%, as shown in Fig. 16 ▸.

The low-temperature X-ray data in Figs. 15 ▸(*a*) and 15 ▸(*b*) show that for dilute (*x* ≤ 2.0%) BZN concentrations BaTiO_3_-like transitions occur, but they are significantly more diffuse and shifted to lower temperatures, relative to BaTiO_3_. Near *x* = 3.0%, the low-temperature *R*3*m* phase is no longer present and a trace tetragonal phase persists to 20 K, as seen in Fig. 15 ▸(*c*). The extinction of the *R*3*m* phase and persistence of *P*4*mm* down to 20 K indicates that no significant temperature-dependent phase transitions take place for *x* > 3.0%. Compositions 3.6 < *x* < 4.0% [Fig. 13 ▸ and Figs. 15 ▸(*d*)–15 ▸(*f*)] show little change in phase abundance with temperature, with the diffracted intensity attributed to *P*4*mm* and *Amm*2 approximately equal for compositions *x* = 3.6% and *x* = 4.0% at *T* < 260 K. Composition *x =* 3.9% shows consistently higher *Amm*2 intensity at all temperatures below *T*_m_ and is the composition with the maximum dielectric polarization and piezoelectric coefficients (Marshall *et al.*, 2020 ▸) at 295 K.

An overall trend in diffracted intensity with temperature for all compositions is that *P*4*mm* decreases at the expense of *Pm*3*m* and *vice versa* near *T*_m_ for compositions *x* ≥ 4.0%. The neutron data, as shown in Fig. 13 ▸(*b*) for *x* = 4.0%, indicate that the *Pm* 3 *m* phase is present at *T* < *T*_m_ for the first time for *x* ≥ 4.0%. Another trend for compositions *x* < 5.0% is that there is an orthorhombic-dominated high-temperature phase near *T*_m_, as shown in Fig. 16 ▸. For *x* ≥ 4.0%, the high-temperature *Amm*2-rich phase coincides with the emergence of the cubic phase, peaking near *T*_m_.

For compositions *x* ≤ 3.9%, the *Amm*2-rich phase emerges before *T*_m_, as seen in Figs. 13 ▸(*a*) and 13 ▸(*b*) and Fig. 15 ▸(*a*)–15 ▸(*e*). Composition *x* = 3.9% in Fig. 15 ▸(*e*) is *Amm*2 dominated for all *T* < *T*_m_ and therefore does not have a discrete *Amm*2-rich region. Another observation concerning *T*_m_ and phase abundance is that, for compositions 1.0 < *x* ≤ 4.0%, *T*_m_ coincides with the point where the intensities of *P*4*mm* and *Pm* 3 *m* are equal, with the exception of *x* = 3.9%. For *x* > 4.0%, *T*_m_ does not necessarily coincide with the emergence of the cubic phase since the tetragonal intensity is low over 20 < *T* < 400 K. The low-temperature phase abundance for *x* = 4.5% and *x* = 5.0% [Figs. 15 ▸(*g*) and 15 ▸(*h*)] combined with lattice parameter data from Fig. 14 ▸(*c*) indicates a weak or absent temperature dependence on the ferroelectric limit near 5.0%.

## 7. Conclusions   

### 7.1. Bi(Zn_2/3_Nb_1/3_)O_3_ in the BaTiO_3_ host   

Dilute additions of BZN have a significant impact on the structure and dielectric properties of the BaTiO_3_ host. Initial additions have the effect of stabilizing the lower-temperature *Amm*2 phase present in the BaTiO_3_ host at 295 K alongside *P*4*mm*.

In contrast to previous studies (Wu *et al.*, 2017 ▸), *Amm*2 was identified as the secondary polar phase owing to the lack of asymmetry of the (222)_c_ peak, as seen in Figs. 1 ▸(*b*), Fig. 2 ▸ and Fig. 3 ▸, and owing to the fact that the *P*4*mm*–*Amm*2 phase transition takes place at around 270 K. BZN addition has the effect of simultaneously lowering the phase-transition temperature and increasing the temperature range of transition at *T* < *T*_m_, making the presence of *Amm*2 alongside *P*4*mm* possible at 295 K. The drop in *T*_m_ and the diffuse nature of the phase transitions are consistent with other studies of relaxor addition in perovskite ferroelectrics (Beuerlein *et al.*, 2016 ▸; Pramanick *et al.*, 2018 ▸). The net result is that well defined temperature-dependent phase transitions are extinct by 2.0 < *x* < 3.0%, as seen in Figs. 13 ▸ and 14 ▸. The structural distortion induced by BZN addition in BaTiO_3_ also has significant effects on the dielectric properties and establishes a pseudocubic-like long-range ferroelectric order over the composition range 3.8 < *x* < 5.0%, where the dielectric and piezoelectric maxima are observed for this system (Marshall *et al.*, 2020 ▸).

### 7.2. Lattice parameters, structural distortion and dielectric properties   

Dilute (1.0 < *x* < 3.0%) additions of BZN to BaTiO_3_ caused an initial contraction in *c*_T_/*a*_T_ which remained stable up to *x* > 3.0%, as seen in Figs. 1 ▸(*a*) and 6 ▸. The orthorhombic phase is detected at *x* = 1.0%, which also coincides with the maximum values of the lattice parameters. The lattice parameters rapidly converge as seen in Fig. 7 ▸. Both polar phases show a striking convergence at *x* = 3.9%, as seen in Figs. 6 ▸ and 7 ▸ for the *P*4*mm* and *Amm*2 phases, respectively, with a narrow compositional minimum in *c*_T_/*a*_T_ and a similar, less well defined, contraction for the orthorhombic *a*_O_, *b*_O_ and *c*_O_ parameters. The resulting pseudocubic-like long-range order is coincident with the maximum dielectric coefficients in this system.

High dielectric coefficients are usually associated with distortions in the crystal structure and, in the case of relaxors, chemical instability (Cross, 1987 ▸) between the PNRs and the host. Investigating the atom parameter displacement with BZN concentration gives some insight into why the long-range order is at its most pseudocubic before the appearance of the paraelectric phase at 295 K, as seen in Figs. 8 ▸ and 9 ▸ for *P*4*mm* and *Amm*2. Both X-ray and neutron data showed that the *B*-site atoms in both polar phases showed a decrease and local minimum in *z* position over 3.8 < *x* < 4.0%, although the uncertainties were generally higher in this region. The O1 site did not show significant changes in position with composition for either *P*4*mm* or *Amm*2 but did show very high uncertainty for 3.8 < *x* < 5.0%, indicating some degree of instability in the O1 position. The O2 positions in *P*4*mm* and *Amm*2 followed the trends in *B*-site position, with a decrease in *z* position and local minimum in *P*4*mm* and in *y* and *z* for *Amm*2. For 4.0 < *x* < 5.0%, the *B*-site and O2 positions were observed to return to *z*-position values (and *y* position for *Amm*2) close to that of the BaTiO_3_ host for *x* ≥ 5.0%. Even with the uncertainties taken into account, it is apparent that atom positions coupled with the pseudocubic appearance of the dominant *Amm*2 phase drive the strong ferroelastic coupling and high dielectric coefficients characteristic of the region 3.8 < *x* < 4.0%. The relaxation of the atom positions for *x* > 4.0% coincides with the break-up in ferroelectric coupling, marked by the sudden drop in *d*_33_ (Marshall *et al.*, 2020 ▸), decline in *Amm*2 intensity, and coexistence of the *Pm* 3 *m* phase at 295 K and *x* ≥ 4.0%.

### 7.3. The region 3.9 < *x* < 5.0% – the ferroelectric-to-relaxor transition region and beyond   

The ferroelectric-to-relaxor transition of the *x*BZN-(1−*x*)BT system has been identified for 4.0 < *x* < 5.0% from previous studies of this system (Paterson *et al.*, 2015 ▸; Wu *et al.*, 2016 ▸, 2017 ▸; Chen *et al.*, 2015 ▸; Marshall *et al.*, 2020 ▸). The present study shows the structural origins of both the dielectric and piezoelectric peaks near *x* = 4.0% and the break in ferroelectric order at *x* ≥ 4.0%, preceding the ferroelectric limit at *x =* 5.0% as seen in Fig. 10 ▸ and Table 4 ▸. The dielectric coefficients for 5 and 640 kHz remained high up to *x* = 6.0%, but the 1 Hz *ɛ*_m_ closely followed the trends in polarization, with a sharp drop at *x* = 4.0%. The loss of ferroelectric long-range order at *x* > 4.0% would affect dielectric polarization and *d*_33_ with the break in ferroelectric coupling, and a similar effect is observed for the 1 Hz dielectric constant, which appears to be more strongly coupled with long-range ferroelectric order as for *P*_max_. The higher-frequency ɛ_m_ values are significantly lower than ɛ_m_ but are less affected by the loss of ferroelectric long-range order, since dipole rotation will be a more significant contributor to the dielectric constant with increasing frequency (Wu *et al.*, 2016 ▸; Chen *et al.*, 2015 ▸).

The structural evidence for ferroelectric decoupling is given by the sudden change in crystal structure between *x* = 3.9% and *x* = 4.0% from X-ray and neutron data, as seen in Figs. 3 ▸ and 6 ▸. Composition *x* = 4.0% marks the point where further BZN addition results in sufficient structural distortion from PNRs that long-range ferroelectric order is no longer present at 295 K. There is sufficient remaining ferroelectric order at *x* > 4.0% that the polarization and *d*_33_ are still high (Marshall *et al.*, 2020 ▸), but there is a significant drop in magnitude from *x* = 3.9% and a large drop in *Amm*2 intensity.

The spike in *c*_T_/*a*_T_ – 1 at *x* = 4.0% is a strong indicator that the ferroelastic coupling in the *x*BZN-(1−*x*)BT system is driven by the PNR-induced *Amm*2 phase. For *x* ≥ 4.0%, after the initial drop at 4.0%, the *Amm*2 intensity returns but the unit cells are more dilated, as for *P*4*mm* in the 4.0 < *x* < 5.0% region. Furthermore, the atom parameters relax to values closer to BaTiO_3_ near *x* = 5.0%. The coexistence of *Pm* 3 *m*, the detection of noticeable dielectric relaxation at *x* ≥ 4.0%, and the fact that the rapid increase in *Pm* 3 *m* content correlates with the decline in *P*4*mm* at 295 K are all consistent with the increasing relaxor behaviour for 4.0 < *x* < 5.0%.

For *x* > 5.0% past the ferroelectric limit, there is still remnant ferroelectric order within the more weakly coupled PNR-dominated paraelectric phase, resulting in the lossy dielectric loops seen by Marshall *et al.* (2020 ▸) to *x* = 8.0%. For *x* > 8.0%, it is assumed that remnant ferroelectric regions are sufficiently small that energy loss during polarization switching is much reduced relative to *x* < 8.0%. Further addition of BZN results in the slim relaxor dielectric loops present for *x* > 10.0%.

## Figures and Tables

**Table 1:** Compositional uncertainty for X-ray and neutron *x*BZN-(1−*x*)BT samples

X-ray samples/ceramics		Neutron samples	
Mol%	±	Mol%	±
0.0	0.000	–	–
1.0	0.050	–	–
2.0	0.050	–	–
3.0	0.051	3.0	0.025
3.6	0.032	3.6	0.025
3.8	0.032	–	–
3.9	0.025	3.9	0.025
4.0	0.033	4.0	0.025
4.2	0.025	4.2	0.025
4.5	0.033	4.5	0.025
5.0	0.033	5.0	0.025
6.0	0.051	6.0	0.025
7.0	0.051	7.0	0.025
8.0	0.025	8.0	0.025
10.0	0.051	–	–
12.0	0.051	–	–
15.0	0.051	–	–
20.0	0.052	–	–

**Table 2:** Source data and parameters for *x*BZN-(1−*x*)BT models and impurity phases

				Lattice parameters (Å, °)										
PDF/reference	Formula	Space group	SG No.†	*a*	*b*	*c*	α	β	γ	*z*	ρ (g cm^−3^)	*V* (Å^3^)	*T* (K)	Comments
00-005-0626	BaTiO_3_	*P*4*mm*	99	3.994	3.994	4.038	90	90	90	1	6.010	64.4	295	Polar phase
01-083-8301	BaTiO_3_	*Amm*2	38	3.996	5.691	5.677	90	90	90	2	5.999	129.1	295	Polar phase
01-085-0368	BaTiO_3_	*R*3*m*	160	5.651	5.651	6.948	90	90	120	3	6.122	192.2	183	*T* < 250 K
01-070-9165	BaTiO_3_	*Pm* 3 *m*	221	4.009	4.009	4.009	90	90	90	1	6.010	64.5	300	Paraelectric phase
01-083-4011‡	Pb(Zr_0.52_Ti_0.48_)O_3_	*Cm*	8	5.669	5.669	4.020	90	90.92	90	2	6.071	133.6	295	*Cm* model trial
04-012-4418	BaTi_2_O_5_	*A*2/*m*	12	9.41	3.94	16.89	90	103	90	–	4.40	610.2	295	Impurity
O’Bryan *et al.* (1974 ▸)	BaTi_5_O_11_	*P*2_1_/*n*	11	7.66	9.30	6.46	90	98.43	90	6	4.42	2210.6	295	Impurity
ICSD 280159	Ba_3_Ti_10_O_16_	*C*1_2_/*m*_1_	12	19.90	11.47	9.92	90	109.4	90	–	4.83	2136.5	295	Impurity – neutron only

† SG: space group.

‡ Basis for *Cm* for *x*(BZN)-(1−*x*)BT with Bi/Ba at the *A* site and Ti/Zn/Nb at the *B* sites.

**Table 3:** Summary of GoF for X-ray and neutron powder diffraction at 295 K from tested model fits Note, for compositions *x* > 4.0%, *Pm* 3 *m* was included in all models.

	X-ray, GoF = *R*_wp_/*R*_exp_			Neutron, GoF = *R*_wp_/*R*_exp_		
BZN mol%	*P*4*mm* + *Amm*2	*P*4*mm * + *Pm* 3 *m*	*P*4*mm* + *Cm*	*P*4*mm* + *Amm*2	*P*4*mm * + *Pm* 3 *m*	*P*4*mm* + *Cm*
0.0	2.17	2.17	2.17	–	–	–
1.0	3.29	4.60	2.47	–	–	–
2.0	2.47	3.17	2.75	–	–	–
3.0	2.04	2.29	2.10	2.78	3.74	3.68
3.6	2.26	2.66	2.57	2.97	5.12	4.84
3.8	2.04	2.28	2.63	–	–	–
3.9	1.32	1.34	1.54	2.75	2.54	2.87
4.0	1.43	1.52	1.43	2.34	4.01	3.02
4.2	1.23	1.34	1.28	1.86	2.98	2.24
4.5	1.16	1.22	1.17	1.94	3.89	2.77
5.0	1.36	1.82	1.70	1.64	1.89	2.00
6.0	1.37	1.64	1.47	1.88	3.11	3.17
7.0	1.50	1.56	1.60	1.99	3.04	2.65
8.0	1.44	1.49	1.90	2.18	2.81	3.67
10.0	1.50	1.64	1.93	–	–	–
12.0	1.66	–	–	–	–	–
15.0	1.53	–	–	–	–	–
20.0	1.82	–	–	–	–	–

**Table 4:** Composition and *T*_m_ for 5 and 640 kHz

BZN		640 kHz		5 kHz	
Mol%	±	*T*_m_	±	*T*_m_	±
0.0	0.000	400	2	400	2
1.0	0.050	392	2	392	2
2.0	0.050	380	4	376	4
3.0	0.051	357	5	355	5
3.4	0.032	343	2	343	2
3.6	0.031	330	2	330	2
3.8	0.032	327	4	326	4
3.9	0.025	317	6	316	6
4.0	0.013	326	6	325	6
4.5	0.013	312	8	303	8
5.0	0.013	290	8	285†	8
6.0	0.051	290	8	280†	10
7.0	0.051	296	8	270†	10
8.0	0.025	350	8	270†	10
10.0	0.051	305	4	275†	4
12.0	0.051	321	3	294	3
15.0	0.051	353	3	293	3
20.0	0.052	369	3	294	3

† Values of *T*_m_ were interpolated from values reported by Wu *et al.* (2016 ▸, 2017 ▸).
